# Probiotic efficacy and mechanism of a pigeon derived *Ligilactobacillus salivarius* strain in promoting growth and intestinal development of pigeons

**DOI:** 10.3389/fmicb.2025.1584380

**Published:** 2025-05-09

**Authors:** Puze Zhao, Yumei Li, Yuwei Yang, Qingxing Xiao, Ziyi Zhang, Xiaoqing Hong, Hongyu Ni, Zhuxuan Xia, Kun Zhan, Sibao Yang, Yonghong Zhang

**Affiliations:** ^1^College of Animal Science, Jilin University, Changchun, China; ^2^Department of Cardiovascular Medicine, China-Japan Union Hospital of Jilin University, Changchun, China

**Keywords:** pigeon, *Ligilactobacillus salivarius*, production performance, intestinal flora, intestinal transcriptomics

## Abstract

**Background:**

With the gradual rise of antibiotic-free farming practices, the exploration of novel, green, and low-pollution alternatives to antibiotics has become one of the key research focus in the field of agricultural science. In the development of antibiotic alternatives, probiotics, particularly host-associated probiotics, have been found to play a significant role in enhancing the production performance of livestock and poultry. However, research on and application of probiotics specifically for meat pigeons remain relatively underdeveloped.

**Objective:**

To assess and investigate the probiotic efficacy and mechanisms during homologous lactic acid bacteria (LAB) transplant to host-pigeons, LAB strains with good probiotic properties were isolated from the intestinal contents of 28-day-old Mimas pigeons. And then measured the production indexes, intestinal flora, and intestinal transcriptomics of the hosts after instillation of LAB strains.

**Methods:**

A total of 360 at 1-day-old pigeons were randomly divided into four groups and gavaged 0.4 mL *Ligilactobacillus salivarius* S10 with concentration of 0, 10^8^, 10^9^, and 10^10^ CFU/mL, designated as the control group (CG), the low concentration group (LG), the medium concentration group (MG), and the high concentration group (HG), respectively.

**Results:**

The findings revealed that an optimal concentration of 10^9^ CFU/mL *L. salivarius* S10, a dominant strain isolated and screened, enhanced the growth performance and intestinal development of young pigeons. 16S rRNA gene sequencing analysis demonstrated a significant increase in the abundance of *Lactobacillus, Pantoea_A* and *Enterococcus_H* and a significant reduction in the abundance of *Clostridium_T* in the pigeon ileum (*p* < 0.05) under selected concentration treatment. Transcriptomic profiling of the ileum revealed 1828 differentially expressed genes (DEGs) between CG and MG. Notably, DEGs involved in the MAPK signaling pathway, such as *RAF1*, *PDGFRB*, and *ELK4*, were significantly correlated with differential ileal bacteria, suggesting that modulation of intestinal flora can influence the expression of genes related to cell proliferation and differentiation in the ileum, which is potentially important in promoting the growth and development of pigeons.

**Conclusion:**

*Ligilactobacillus salivarius* S10 possesses the potential to be used as a probiotic for pigeons, which can influence the expression of gut development-related DEGs by regulating the intestinal flora, and further improve the growth performance of pigeons. This research provides a scientific foundation for developing pigeon-specific probiotics and promotes healthy farming practices for meat pigeons. Furthermore, it opens new avenues for improving the economic efficiency of pigeon farming.

## 1 Introduction

The pigeon industry, as an emerging specialized economic poultry industry, is moving toward modernization and intensification, and the comprehensive benefits behind it are gradually improving. Farmers must pay special attention to the nutritional content and breeding management of pigeons, in order to improve the efficiency of meat or egg production, while ensuring the healthy development of pigeons (Ji et al., 2022; Wen J. et al., 2022), e.g., avoiding the death of animals or the impairment of poultry product quality due to the invasion of the gastrointestinal tract by some pathogenic bacteria. Reasonable addition of the necessary feed additives, can play a multiplier effect. Previous work has demonstrated that the microbial communities colonizing the gastrointestinal tract of poultry play an important role in metabolism, growth and maintenance of health, which provides new ideas for the development of natural growth promoters (Elokil et al., 2022). Probiotics, as a green, safe, harmless to the human body, and effective control of pathogenic bacteria, emerged as a natural feed additive (Michalak et al., 2021).

Probiotics are defined as active bacteria that colonize an animal’s gut and provide health benefits to the host (Salminen et al., 2021). As a new substitute for antibiotics, probiotics can effectively avoid drug residues, increased drug resistance and harm to human health (Ayalew et al., 2022). The microbial preparations that are more commonly used in animal production broadly include, *Lactobacilli*, *Bifidobacterium*, *Bacillus*, *Enterococcus*, *Pediococcus* and yeast (Seal et al., 2018). There exists 6 families and 38 genera belonging to the taxonomy of Firmicutes phylum, Bacilli class, and Lactobacillales order in the group of lactic acid bacteria (LAB) that are the main members of probiotics (Agriopoulou et al., 2020). The most representative *Lactobacilli* is the largest genus in this group, comprising of 261 species (at March 2020), and is widely used in poultry production by different adding methods (individual cultures or cultures combined with other additives) (Zheng et al., 2020; Deng et al., 2021), which plays a vital role in host’s body growth. For example, *L. salivarius* can secrete protease, lipase and amylase, which can be used to improve the body weight and reduce the feed-to-gain ratio in broiler pullets (Yang et al., 2023). Similarly, supplementation of *Lactiplantibacillus plantarum* in broiler diets increased average daily weight gain and apparent ileal digestibility of crude protein, and significantly increased jejunal villus height and decreased jejunal crypt depth (Vimon et al., 2023). Along these lines, LAB supplementation could be used as a potential means of promoting growth and development in oviparous animals, and the premise for achieving this may be inextricably linked to the development of the intestine. However, different LAB strains in poultry exhibit distinct metabolic properties and physiological functions, the optimal concentration for administering probiotics is strain dependent. Therefore, optimizing the dose of LAB is crucial to balance the benefits of colonization with potential risks, as higher doses do not necessarily guarantee better performance. It was found that the addition of *Lactobacillus casei* at 77 mg/kg in the feed significantly increased the body weight and body weight gain of broilers compared to the for-treatment group, whereas higher doses of *L. casei* (457 mg/kg) were not effective in the growth of broilers (Huang et al., 2004). Additionally, different poultry species have unique physiological characteristics, which result in different responses to LAB. This necessitates targeted research for each species. Pigeons, being late fledglings, rely on parental crop milk for nutrition after birth and cannot eat independently (Wen J. S. et al., 2022). This unique characteristic has led to a scarcity of research on LAB in pigeons. Further research is needed to explore the optimal application of LAB in pigeons and other poultry species, which could provide valuable insights for improving poultry health and production efficiency.

It is important to note that not all LAB qualify as probiotics. Before this can be done, strains need to be fully characterized, including isolation, identification and evaluation of their probiotic properties, such as resistance to digestive enzymes, bile or acids, antagonism against pathogens (Fontana et al., 2013). Although ample studies have been conducted the excellent effect of most commercial milk or food-derived probiotics, due to the host-specificity of probiotics (Duar et al., 2017a), there are still some limitations in the application of this type of probiotics in poultry (Jensen et al., 2011). The ideal and compatible probiotics should come from the same ecological source, so that the probiotics can better adapt to the gastrointestinal conditions of animals (Argyri et al., 2013). Pigeons have a relatively short intestine and their intestinal digestive enzymes are different from those of other poultry (Dong et al., 2012). The pigeon’s diet is often based on high fiber foods such as cereals, and this diet also allows for specific bacterial groups (*Lactobacillus* and *Limosilactobacillus*) to dominate the pigeon gut microbiota to aid in the fermentation of cellulose (Xu et al., 2024), whereas other fowls may have a different dominant microbiome. Consequently, pigeon-derived probiotics exhibit distinct advantages in terms of adaptability, colonization capacity, and functional efficacy. In contrast, non-host-specific probiotic strains may demonstrate reduced effectiveness in pigeons due to these physiological and ecological differences. Additionally, our previous study demonstrated that fecal microbiota transplantation (FMT) of ileal flora from Shiqi pigeons into newborn Mimas pigeons significantly enhanced the growth performance of the pigeons (Ren et al., 2024). *Lactobacilli* were identified as the predominant flora in the intestinal tract of the pigeons, suggesting that LAB have growth-promoting potential for Mimas pigeons. These findings highlight the importance of developing pigeon-specific probiotics to optimize their health and productivity. Based on this, the objective of the present study was to isolate and screen high-quality pigeon-derived LAB from pigeons that have received FMT intestinal contents, evaluate and verify its effects on pigeon production performance, intestinal development and intestinal flora, as well as pay attention to the relationship between the growth development, the intestinal microbiome, and the intestinal transcriptome for revealing the possible mechanisms of LAB supplementation to promote pigeon growth. The findings of this study not only provide excellent strain resources for the future development of pigeons’ special micro-ecological preparations, but also provide precious theoretical basis for the academic community.

## 2 Materials and methods

### 2.1 Animal care

The present experiment was reviewed and approved by the College of Animal Science of Jilin University Ethics Committee (SY202306051).

### 2.2 Isolation of lactic acid bacteria strains

0.1 g ileal contents of three 28-day-old Mimas pigeons that have received FMT from Chengcheng Agricultural Development Co., Ltd. (Liaoyuan, Jilin, China) were collected under sterile conditions as samples and inoculated into 50 mL sterilized MRS broth medium at 37°C for 48 h. Next, 100 μL medium was taken in 900 μL sterile PBS and vortexed evenly, which was regarded as a dilution of 10^−1^. And so on, diluted to 10^−5^. Afterwards, 100 μL of each of the last three diluents was evenly spread on the sterilized MRS agar medium containing 2% CaCO_3_ and incubated at 37°C for 48 h. The colonies with the characteristics of LAB (milky white, smooth, and with calcium-dissolving circle) were selected from the medium with moderate density. Then, they were transferred to tubes containing 10 mL MRS broth at 37°C for 24 h and purified by 3 zone streak plate cultivation on nutrient agar at 37°C for 48 h. Gram-positive and catalase-negative pure colonies can be initially identified as LAB and stored in MRS broth containing 50% sterile glycerol at −80°C for further analysis.

### 2.3 Identification of lactic acid bacteria strains

The strains were cultured overnight in MRS Broth at 37°C, and 3 mL of each bacterial solution were centrifuged at 13400 × *g* for 1 min to enrich the bacteria. The genomic DNA of the stains was extracted by DNA extraction kit (TIANGEN, Beijing, China.), and used as template for PCR amplification to obtain large amounts of 16S rRNA gene. Primers used in amplification were universal primers (synthesized by Comate Bioscience Co., Ltd., Jilin, China.) as follows:

27F (5’-AGAGTTTGATCMTGGCTCAG-3′)
1541R (5’-AAGGAGGTGATCCAGCC-3′)

The PCR reactions were performed under the following conditions: initial denaturation at 95°C for 3 min; 32 cycles of denaturation at 94°C for 15 s, renaturation at 55°C for 15 s, and extension at 72°C for 90 s; final extension at 72°C for 5 min. The PCR products were detected by 1% agarose gel electrophoresis. For sequencing, qualified PCR products were recovered and sent to Comate Bioscience Co., Ltd., Jilin, China. The obtained nucleotide sequences were subjected to BLAST alignment on the NCBI,^1^ and the phylogenetic analysis was drawn using the neighbor-joining algorithm by MEGA 11.0 software.^2^

### 2.4 Antimicrobial activity against pathogens

The ultra-low temperature preserved *Escherichia coli* ATCC 25922, *Salmonella* ATCC 14028 and *Staphylococcus aureus* ATCC 6538 were accessed in LB broth medium to restore their activity, and the concentration of the bacterial solution was adjusted to 10^7^ CFU/mL. At the same time, the isolated strains were inoculated into MRS broth mediums and incubated for 24 h, and the concentration of the bacterial solution was adjusted to 10^8^ CFU/mL. The bacteriostatic effect was determined using Oxford cup method. Each pathogen was taken and added into LB medium at 1%, shaken well and then inverted the plate, after the agar solidified, an Oxford cup (7.8 mm) was placed horizontally on the plate. Add 200 μL of bacterial suspension into the Oxford cup, incubate at 37°C for 12 h, and measure the diameter of the inhibition circle with vernier calipers. Three parallels were made for each sample and the average value was taken as the result.

### 2.5 Growth curve and acid production capacity

Growth curve procedure involves taking the bacterial solution to be tested and inoculating it into sterile MRS broth medium with a 1% inoculum. The mixture is then incubated at 37°C for 24 h. Using uninoculated sterile MRS broth medium as a blank control, samples are taken every 2 h to determine the OD_600_ values. For the acid production curve, the bacterial solution to be tested is inoculated into sterile MRS broth medium with a 1% inoculum. The inoculated medium is incubated at 37°C for 24 h. Samples are collected every 2 h, and their acid production capacity is measured using a pH meter.

### 2.6 Tolerance to gastrointestinal tract conditions

The acid resistance test was conducted using the methods of Wang et al. (2023) with minor modifications. The MRS broth medium was adjusted to pH levels of 3, 4, and 5 using 1 mol/L HCl and NaOH, autoclaved at 121°C for 20 min, and prepared for use. The bacterial solution to be tested was inoculated into these MRS broth media with different pH levels at a 2% inoculum. MRS broth medium without pH adjustment (pH = 6) served as the control. All samples were incubated in a shaking bed at 37°C for 4 h, and OD_600_ values were measured under different pH conditions. The test was repeated three times to calculate the survival rate of the isolated strains, assessing their tolerance to acidic environments.

The bile salt tolerance test followed the methods outlined by Sirisopapong et al. (2023) with slight modifications. MRS broth media with bile salt concentrations of 0.05, 0.1, 0.2, and 0.3% were prepared, autoclaved at 121°C for 20 min, and prepared for use. The bacterial solution to be tested was inoculated into these MRS broth media with different bile salt concentrations at a 2% inoculum volume. MRS broth medium without bile salt served as the control. All samples were incubated at 37°C for 4 h, and OD_600_ values were measured under different bile salt concentrations. The test was repeated three times to determine the tolerance of the isolated strains to bile salts by calculating their survival rate.

For the digestive enzyme resistance test, the bacterial solution was centrifuged at 7155 × g for 10 min at 4°C, the supernatant was discarded, and the cells were washed with PBS buffer three times. The cells were then resuspended in MRS broth medium containing trypsin (1 mg/mL) at pH = 7 to simulate the environment of the strain passing through the small intestine. OD_600_ values were measured at 0 h and 4 h. The test was repeated three times to calculate the survival rate of the isolated strains, assessing their resistance to digestive enzymes.

### 2.7 Experimental animals

Meat pigeon breeding adopted a “2 + 2” production mode, as two young pigeons were raised by a pair of healthy Mimas parents per cage. A total of 360 at 1-day-old Mimas young pigeons that broke their shells at the same time were randomly divided into four treatment groups, with 3 replicates of 30 each. The gavage solution was prepared from 90 mL sterile MRS medium or *L. salivarius* S10-containing MRS medium, and 30 mL cryoprotectant solution (50% sterile glycerol). The four treatments were: the control group (CG, gavaged with sterile solution), the low concentration group (LG, gavaged with bacterial concentration of 10^8^ CFU/mL solution), the medium concentration group (MG, gavaged with bacterial concentration of 10^9^ CFU/mL solution) and the high concentration group (HG, gavaged with bacterial concentration of 10^10^ CFU/mL solution). Concentrations were chosen based on previous studies that successfully used similar concentrations of supplemental probiotics in poultry experiments (Wang et al., 2020). Young pigeons in each group were gavaged with 0.4 mL solution by a 1 mL sterile syringe at 10:00 A.M. daily after they were fed by parental pigeons from day 1 to 7. According to the nutritional requirements recommended by NRC (Dale, 1994), the four groups of pigeons were fed with the same basic diet including pellet diet and raw grain according. The nutrient values of the basal diets were completely referenced to the experiments of Ren et al. (2024). During the feeding trial period, pigeons were free to drink water, feed grain and health care sand. All suckling pigeons were vaccinated against Newcastle disease on day 7 and pigeon pox on day 14. Additionally, the pigeon lofts were disinfected regularly to ensure hygiene and sufficient light. The feeding experiment was conducted over a period of 28 days.

### 2.8 Measurement of production indexes and sample collection

The body weight of each group was weighed on the 1st, 7th, 14th, 21st, and 28th days of the experiment, and the body size of each group was measured at the end of the experiment. Subsequently, refer to AVMA (Leary et al., 2020), when the live pigeons were rendered unconscious by electrocution, they were immediately slaughtered and exsanguinated. The dressed weight (the weight of poultry after bloodletting, removal of feathers, foot cuticle, toe shell and beak shell), half-eviscerated weight (the weight of dressed weight minus its trachea, esophagus, crop, intestine, spleen, pancreas, gall bladder, reproductive organs, stomach contents and keratin) and eviscerated weight (the weight of half-eviscerated weight minus the weight of heart, liver, muscle stomach, glandular stomach, lung and abdominal fat) of the pigeons were recorded. Additionally, the breast muscle on both sides were removed and weighed. On day 28, samples were randomly selected from the CG and MG. Three pigeons’ ileal intestinal segments per group were collected for the analysis of intestinal morphology, and also stored in liquid nitrogen for further RNA-seq analysis. Six pigeons’ ileal contents per group were immediately collected for gut bacterial 16S rRNA gene sequencing analysis.

### 2.9 Intestine morphological analyses and observation

Each pigeon was collected about 1 cm of ileal intestinal segment at the same site, which was fixed in 4% paraformaldehyde for 48 h. After that, it was subjected to water flushing, gradient alcohol dehydration, xylene transparent and paraffin embedding, and sections of 5 μm thickness were stained with routine hematoxylin–eosin (HE) staining, and finally sealed with neutral resin. The villus height and crypt depth of each specimen were measured under an optical microscope (14.0 ×) using the Slide Viewer image analysis system (3DHISTECH Ltd., Budapest, Hungary) for five intact and well oriented villi (i.e., 15 villi in each group), and the values of villus height/crypt depth (VH/CD) were calculated.

### 2.10 DNA preparation and ileal microbiota analysis

Microbial genomic DNA was extracted from the collected ileal contents by a DNA extraction kit (Omega Bio-Tek, Norcross, GA, USA) according to manufacturer’s instructions. The quality of DNA was assessed by the Nanodrop NC2000 spectrophotometer (Thermo Fisher Scientific, Waltham, MA, USA) and agarose gel electrophoresis. The V3V4 hypervariable region of bacterial 16S rRNA amplified by PCR using forward primer 338F (5’-ACTCCTACGGGAGGCAGCA-3′) and reverse primer 806R (5’-GGACTACHVGGGTWTCTAAT-3′). Meanwhile, the barcode sequence unique to each sample was attached into the primers for multiplexing. The PCR amplicons were purified by Vazyme VAHTSTM DNA Clean Beads (Vazyme, Nanjing, China), and Quant-iT PicoGreen dsDNA kit (Invitrogen, Carlsbad, CA, USA) was used for quantitative analysis. The amplicons were sequenced by the Illumina NovaSeq platform (2 × 250 bp paired-end run) and NovaSeq 6,000 SP Reagent Kit at Personal Biotech Co., Ltd., Shanghai, China.

The QIIME2 software package (version: 2019.4) was employed for analyses of the obtained sequencing data. Raw sequence data were demultiplexed using the demux plugin followed by primer cutting with cutadapt plugin. The imported paired sequences were quality filtered, denoised, and merged through the DADA2 plugin to generate the amplicon sequencing variants (ASVs), and then the chimeric sequences and singleton ASVs were removed. Subsequently, the Greengenes database^3^ was used to classify and annotate non-singleton ASVs. The taxa plugin was invoked to visualize the composition distribution of each group at the phylum and genus level. The diversity plugin was invoked for a variety of analyses, including alpha diversity analysis (Chao1, Observed species, Shannon and Simpson), sparse curves drawing, and beta diversity analysis based on the Weighted UniFrac algorithm. Among them, beta diversity analysis was presented by principal coordinate analysis (PCoA) and tested for significance using the analysis of similarities (ADONIS) method. The unique and shared ASVs between two groups were calculated by the VennDiagram R package. Linear discriminant analysis effect size (LEfSe) and Wilcoxon test were used to screen significantly enriched taxonomic units from all taxonomic units from phylum to genus (LDA > 2, *p* < 0.05).

### 2.11 Library construction and bioinformatics analyses of RNA-seq data

Total RNA was extracted from ileum samples using the TRIzol (Invitrogen, Carlsbad, CA, USA) method following manufacturer’s instructions. The quality of RNA was measured using the Nanodrop NC2000 spectrophotometer (Thermo Fisher Scientific, Waltham, MA, USA), agarose gel electrophoresis, and the Bioanalyzer 2,100 system (Agilent, Palo Alto, CA, USA). Sequencing libraries were generated using the NEBNext Ultra RNA Library Prep Kit for Illumina (New England Biolabs, Ipswich, MA, USA). In brief, the mRNA was first enriched by oligo-attached magnetic beads and segmented in an Illumina proprietary fragmentation buffer. Subsequently, the first-strand cDNA was synthesized using random oligonucleotides and SuperScript II, and the second-strand cDNA was synthesized using DNA Polymerase I and RNase H. After that, the processes including cDNA purification, addition of sequencing connector and PCR amplification were performed successively to complete the cDNA library construction. Finally, Agilent Bioanalyzer 5,400 system was used to evaluate the quality of the library, and the eligible library was sequenced on the Illumina NovaSeq 6,000 platform of Novogene Co., Ltd., Beijing, China.

Fastp software was used to filter the sequenced raw reads to remove the sequencing data contained a number of adaptors and low-quality reads, and obtain high-quality clean reads for further analysis. Then, clean reads were mapped to referential pigeon genome using STAR software and the comparison rates were calculated. Next, by DESeq2 software, counts of each sample gene were normalized and principal component analysis (PCA) was performed. Meanwhile, |log2FoldChange| > 1 and *p* < 0.05 were used as the screening criteria for the differentially expressed genes (DEGs). DAVID^4^ was used to conduct gene ontology (GO) functional enrichment analysis and KOBAS^5^ was used to conduct kyoto encyclopedia of genes and genomes (KEGG) pathway enrichment analysis for the selected significant DEGs. “*p* < 0.05” was set as the threshold for significant enrichment in GO and KEGG analyses. All visualizations of the transcriptome were plotted by SRplot.^6^

### 2.12 Quantitative real-time PCR validation

Quantitative Real-time PCR (qRT-PCR) was used to verify the mRNA expression level of the random selections of DEGs in ileum, and the information on the primers used in this study was shown in Table 1. First of all, the cDNA for qRT-PCR validation was reverse transcribed from total RNA by All-In-One 5 × RT MasterMix (Applied Biological Materials, Richmond, BC, Canada.), and the entire reaction was performed in the CFX Duet system (Bio-Rad, Shanghai, China.). The total volume of the qRT-PCR reaction was 20 μL, consisting of 10 μL Blastaq 2 × qpcr Master Mix (Applied Biological Materials, Richmond, BC, Canada), 7 μL RNase-free H_2_O, 2 μL cDNA, and 0.5 μL of each primer. Each cDNA was repeated three times, with the columba livia *β-actin* gene as the reference. The cycling conditions were as follows: 95°C for 3 min, then 40 cycles of 95°C for 15 s, and then 60°C for 1 min. Finally, the formula 2^−ΔΔCt^ was used to calculate the relative mRNA expression of the validated genes, and the results were compared with fragments per kilobase of transcript per million fragments mapped (FPKM) values from the transcriptomic data to confirm the accuracy of RNA-seq.

**Table 1:** Primer sequences used for qRT-PCR.

Gene^1^	Accession number	Primer nucleotide sequence (5′ → 3′)^2^	Product length (bp)	Reference^3^
*PCK1*	XM_005499615	F: GGTGATGATATTGCCTGGAT R: GTGCTACATTGGTGAAGATG	160 bp	Current study
*TMPPE*	XM_065055142	F: CCTTCTTATCTTCTTCCTCTTC R: GCTGTTCTGCTGTTCTTG	108 bp	Current study
*TSPAN1*	XM_005501009	F: GCAAGTGTCTCCTGATTATG R: GTGAAGTCTGTGTAGTTAGTC	232 bp	Current study
*PDGFRB*	XM_065029783	F: CTGGTCTGCTGGAGGTAA R: GTGAGGTTGCTGATGAAGA	193 bp	Current study
*RAF1*	XM_065074431	F: ACTGTATCTCACCTACTATCG R: AGCATCTGTATTCCAATCCA	278 bp	Current study
*ELK4*	XM_065038630	F: GAAGGAGAAGTCGCAGTC R: TTGACGCTGGAAGAGTTG	108 bp	Current study
*β-actin*	AB618546	F: GCGTGACATCAAGGAGAA R: GATACCAACAGATTCCATACC	196 bp	Current study

^1^*PCK1*, phosphoenolpyruvate carboxykinase 1; *TMPPE*, transmembrane protein with metallophosphoesterase domain; *TSPAN1*, tetraspanin 1; *PDGFRB*, platelet derived growth factor receptor beta; *RAF1*, Raf-1 proto-oncogene, serine/threonine kinase; *ELK4*, ETS transcription factor ELK4. ^2^\u00B0F, forward primer; R, reverse primer. ^3^Current study represents the sequence was designed independently by Primer Premier 6.0 (PREMIER Biosoft, San Francisco, CA, USA).

### 2.13 Statistics and analysis

Statistical analysis was performed using SPSS Statistics 26.0 software (SPSS, Inc., Chicago, IL, USA). One-way ANOVA was performed on production performance data, and Duncan method was used to make multiple comparisons. Student’s t-test was performed on intestinal morphological data and gene expression data. “*p* < 0.05” was used as the criterion to determine the significant difference. The results were expressed as “mean ± standard deviation (SD)” and visualized using GraphPad Prism 8 software (San Diego, CA, USA). All Spearman’s correlation analyses were examined by Genescloud.^7^ Significant differences at *p* < 0.05, 0.01 and 0.001 were indicated as *, ** and ***, respectively.

## 3 Results

### 3.1 Isolation and molecular biological identification of lactic acid bacteria strains

**Figure 1:**
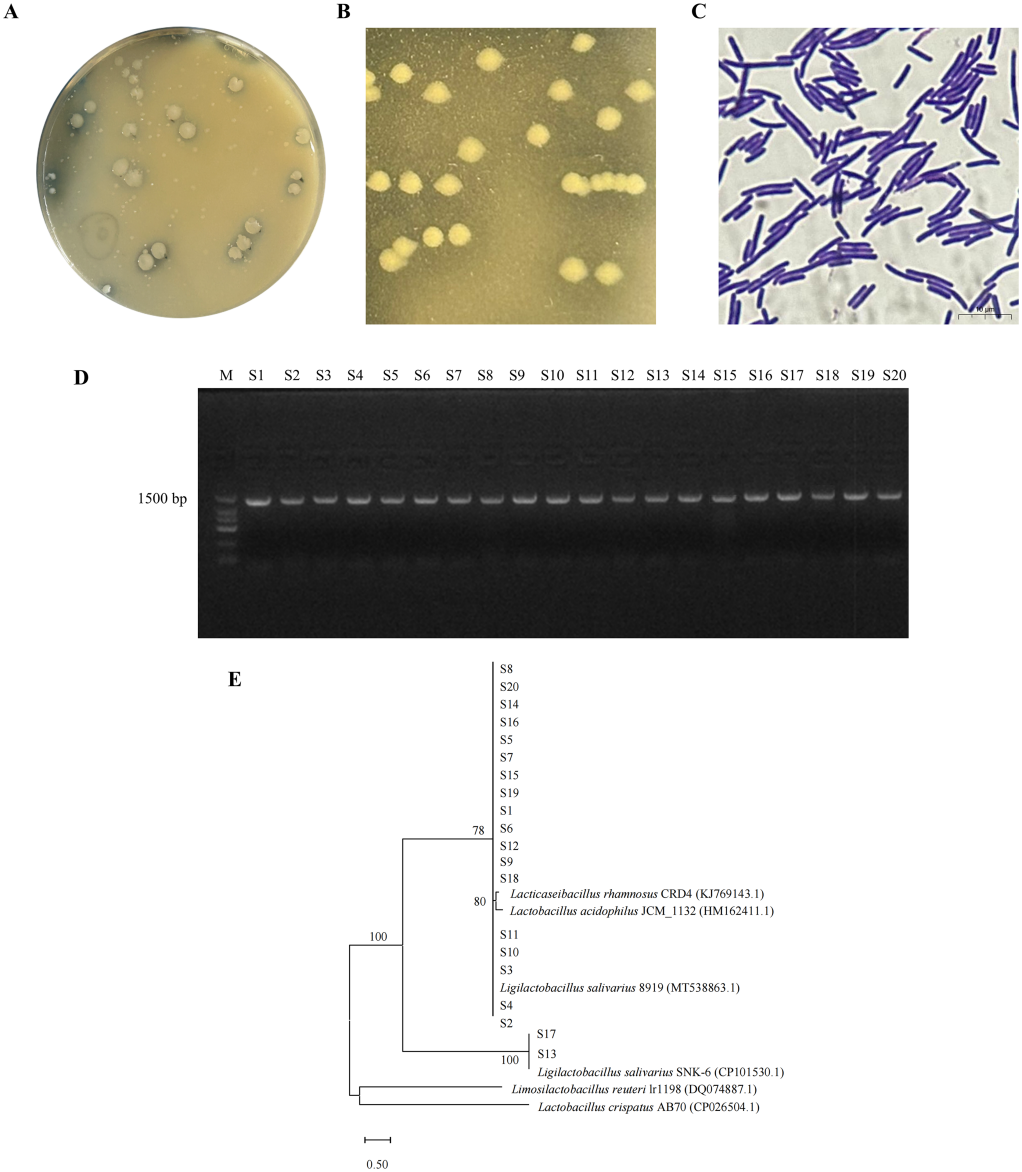
Isolation, purification and identification of LAB strains. **(A)** MRS agar medium containing 2% CaCO_3_ at the dilution of 10^−4^. **(B)** Depict the colony morphology of the isolated strains after three times of purification. **(C)** Depict the Gram staining of the isolated strains after three times of purification. **(D)** S1–S20 represents the results of 16S rRNA gene amplification for isolated strains. **(E)** Phylogenetic tree of isolated strains.

After enrichment culture of pigeon gut contents, serial dilutions were spread on plates. After 48 h, the colony density on the plate with a dilution factor of 10^−4^ was moderate (Figure 1A). A total of 20 strains were isolated and purified from all colonies, and named serially from S1 to S20. The isolated strains were round, milky white, smooth surface, and showed clear calcium-dissolving circle around the colonies on MRS medium supplemented with 2% CaCO_3_ (Figure 1B). Gram staining revealed positively stained, rod-shaped, no spores, rounded at both ends, forming short chains in pairs or several forming long chains (Figure 1C). Bacteria shown in Figures 1B,C are S10. The morphological results of all isolated strains are shown in Supplementary Figures 1, 2. The target fragments obtained by agarose gel electrophoresis were all bands of about 1,500 bp, which was consistent with the length of the 16S rRNA gene (Figure 1D). The results of species identification showed that strains S7 and S13 were closely related to *L. salivarius* SNK-6 (CP101530.1), and all other strains were closely related to *L. salivarius* 8,919 (MT538863.1) (Figure 1E).

### 3.2 Inhibition of pathogenic bacteria by isolated strains

As shown in the Table 2, the twenty isolated strains had different degrees of inhibitory effect on *E. coli*, *S. aureus* and *Salmonella*. Among them, S10 had the strongest inhibitory ability against *Salmonella*, and the diameter of the inhibition zone reached 18.76 mm. S7 had the strongest inhibitory ability against *E. coli*, and the diameter of the inhibition zone reached 16.04 mm. S19 had the strongest inhibitory ability against *S. aureus*, and the diameter of the inhibition zone reached 15.71 mm. S5 and S11 were strong antibacterial effects on two of the three pathogenic bacteria, which also showed excellent bacteriostatic performance. Overall, most of the strains showed better inhibitory effects against *Salmonella* than against *E. coli* and *S. aureus*. S5, S7, S10, S11 and S19 showed outstanding performance and should be selected for further screening (The image of bacteriostatic activity is shown in Supplementary Figure 3).

**Table 2:** The antibacterial activity of isolated strains against different pathogenic bacteria (mm)^1^.

Isolated strains	Pathogenic bacteria		
	*E. coli*	*S. aureus*	*Salmonella*
S1	13.86 ± 0.70	13.71 ± 0.96	16.44 ± 1.13
S2	13.82 ± 0.73	12.77 ± 0.30	15.87 ± 0.47
S3	14.04 ± 0.52	14.09 ± 0.36	15.46 ± 1.92
S4	14.17 ± 0.67	13.17 ± 1.85	13.52 ± 1.24
S5	15.99 ± 0.67	14.36 ± 0.76	17.89 ± 0.95
S6	14.29 ± 0.21	11.62 ± 0.30	14.19 ± 0.26
S7	16.04 ± 0.75	13.97 ± 0.48	18.51 ± 0.52
S8	13.47 ± 0.27	14.59 ± 0.68	17.92 ± 1.14
S9	13.85 ± 0.81	11.89 ± 0.86	14.77 ± 0.99
S10	15.60 ± 0.54	14.76 ± 0.78	18.76 ± 0.55
S11	15.47 ± 2.61	13.06 ± 1.02	15.40 ± 0.33
S12	13.17 ± 0.69	12.89 ± 0.08	15.75 ± 0.45
S13	14.42 ± 0.61	13.91 ± 1.37	16.77 ± 0.13
S14	14.67 ± 0.36	14.75 ± 0.20	16.84 ± 0.79
S15	13.33 ± 1.05	12.11 ± 0.18	18.20 ± 1.31
S16	11.84 ± 0.62	14.22 ± 0.32	16.92 ± 0.66
S17	12.67 ± 0.73	12.08 ± 0.60	16.48 ± 0.36
S18	13.28 ± 0.69	13.14 ± 0.26	15.76 ± 0.71
S19	14.21 ± 0.23	15.71 ± 0.17	17.51 ± 0.83
S20	13.50 ± 0.13	13.16 ± 0.10	14.44 ± 0.63

^1^The antibacterial activity is expressed by the diameter of the inhibition zone. < 10 mm indicates weak antibacterial effect; 10–15 mm indicates moderate antibacterial effect; > 15 mm indicates strong antibacterial effect. The data are expressed as mean ± SD (*n* = 3).

### 3.3 *In vitro* characterization of isolated strains

**Figure 2:**
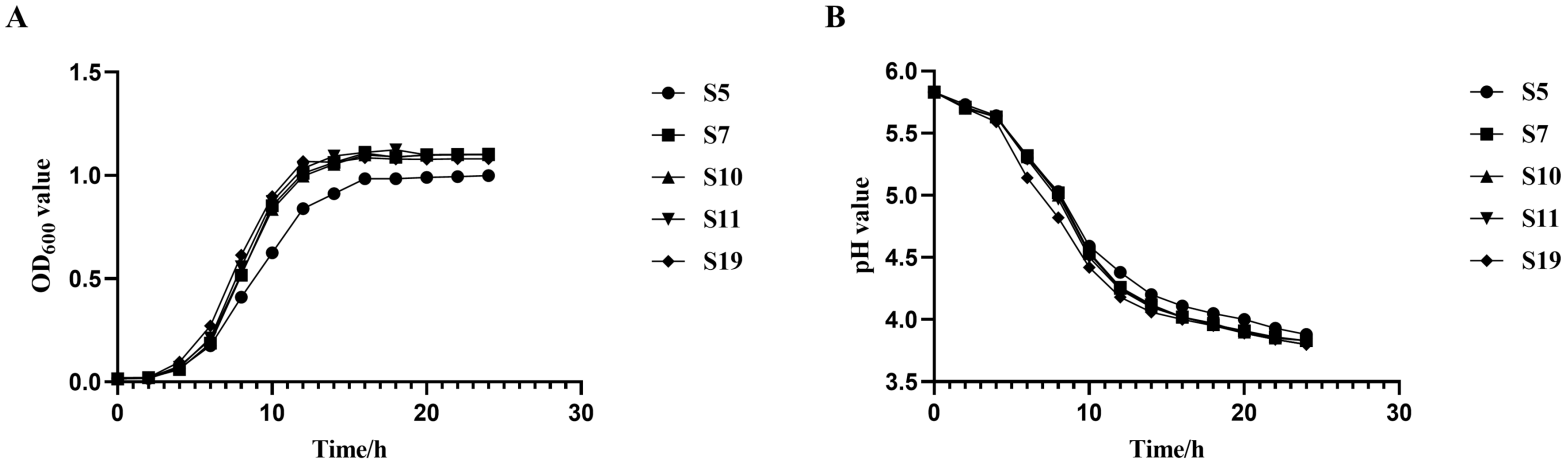
Growth and acid-producing capacity of isolated strains. **(A)** Growth curve. **(B)** Acid-producing ability.

The five isolated strains were in the growth retardation period from 0 to 4 h, and the pH values did not change much. At about 4 h, the strains entered the logarithmic growth period, and the pH values decreased rapidly, but the proliferation rate of S5 was not as fast as the other four strains. At about 16 h, the strains entered the stabilization period, the OD_600_ values gradually stabilized, and the pH values decreased slowly. After 24 h of incubation, the pH values of all five strains decreased from 5.8 to about 3.8, which showed a strong acid production capacity (Figures 2A,B). Interestingly, not only did these strains demonstrate robust acid production, but their survival rates under varying environmental conditions also varied significantly. The results showed that the survival rate of strains showed a decreasing trend with either decreasing pH or increasing bile salt concentration. The results showed that the survival rate of strains showed a decreasing trend with either decreasing pH or increasing bile salt concentration (Table 3). While at pH = 3, All isolated strains were able to survive for 4 h, but S11 showed a higher survival rate of 25.29%, followed by S10 with 19.64%. However, the situation changed in different concentrations of bile salt environments. S10 showed better survival than the other strains, including S11, on the whole. In addition to acidic and bile salt conditions, the digestive enzyme resistance characteristics of the isolated strains were examined. The results showed that all five strains showed good resistance to trypsin after 4 h of incubation in a simulated small intestine environment, with survival rates higher than 90% (Table 3). This transition in focus from their growth and acid production characteristics to their survival under stressful conditions highlights the versatility and adaptability of these strains.

**Table 3:** The tolerance of isolated strains to acid, bile salts and digestive enzymes (%)^1^.

Isolated strains	pH value			Bile salts			Trypsin
	pH = 5	pH = 4	pH = 3	0.05%	0.10%	0.20%	1 mg/mL
S5	42.43 ± 1.44	19.14 ± 1.00	15.65 ± 0.75	25.50 ± 0.70	10.28 ± 1.02	6.20 ± 0.32	95.09 ± 1.20
S7	42.09 ± 0.52	23.43 ± 0.84	15.15 ± 0.41	23.87 ± 0.67	11.81 ± 0.42	6.35 ± 0.14	94.32 ± 1.22
S10	52.25 ± 1.17	28.29 ± 0.47	19.64 ± 1.81	31.21 ± 0.75	15.02 ± 0.11	6.80 ± 0.26	92.03 ± 0.96
S11	50.54 ± 0.58	37.84 ± 0.46	25.29 ± 1.97	32.62 ± 0.76	11.05 ± 0.61	5.07 ± 0.20	90.07 ± 0.51
S19	54.24 ± 1.20	22.09 ± 0.63	17.87 ± 0.98	23.30 ± 0.18	12.44 ± 0.59	6.48 ± 0.30	93.37 ± 0.61

^1^The data are expressed as mean ± SD (*n* = 3).

### 3.4 The effect of *Ligilactobacillus salivarius* S10 on on growth performance of pigeons

Combining the results of the above tests, *L. salivarius* S10, which has good tolerance to acid, bile salts and digestive enzymes, and has the strongest inhibitory effect on *Salmonella*, was selected as a candidate strain and configured as a bacterial solution to be instilled into the newborn pigeons. To evaluate the impact of this bacterial solution on the growth of the newborn pigeons, various groups were established, including a control group (CG) and groups receiving low (LG), medium (MG), and high (HG) concentrations of *L. salivarius* S10. Initially, at 1 day of age, there was no significant difference in body weight among all groups (*p* > 0.05). This suggests that the initial conditions for all pigeons were comparable.

However, the body weight of MG was slightly higher than that of CG, LG and HG at 7, 14 and 21 days of age, but the difference was not significant (*p* > 0.05). At the age of 28 days, compared with CG, the body weight of MG and HG was significantly increased by 40 and 25.5 g, respectively (*p* < 0.01). Meanwhile, the weight of pigeons in MG was also significantly higher than that in LG (*p* < 0.01) (Table 4). In terms of keel length, MG was significantly increased compared with CG and LG (*p* < 0.01), but there was no significant difference compared with HG (*p* > 0.05) (Table 5). Compared with CG and LG, the dressed weight, half-eviscerated weight and eviscerated weight of pigeons in MG were significantly increased (*p* < 0.05). The breast muscle weight in MG and HG was significantly higher than that in CG (*p* < 0.05). In each index, MG showed a higher advantage (Table 6).

**Table 4:** Different concentrations of *L. salivarius* S10 affect the body weight of pigeons at various time-points (g)^1^.

Weight	CG	LG	MG	HG	*p* value
1 d	15.6 ± 1.6	15.9 ± 2.1	15.6 ± 1.6	15.7 ± 1.6	0.988
7 d	150.4 ± 23.0	152.0 ± 18.1	160.2 ± 27.0	159.1 ± 26.1	0.761
14 d	351.0 ± 40.0	357.0 ± 38.4	383.4 ± 17.0	383.0 ± 38.4	0.112
21 d	539.8 ± 28.6	530.2 ± 22.3	550.0 ± 22.1	548.4 ± 34.8	0.407
28 d	535.7 ± 16.0^C^	551.3 ± 18.5^BC^	575.7 ± 12.8^A^	561.2 ± 22.8^AB^	< 0.001

^1^The data are expressed as mean ± SD (*n* = 90). ^AB^ Values within a row with no common superscripts differ significantly (*p* < 0.01).

**Table 5:** Different concentrations of *L. salivarius* S10 affect the 28-day body size of pigeons (mm)^1^.

Items	CG	LG	MG	HG	*p* value
Chest depth	69.19 ± 3.47	68.66 ± 3.61	72.36 ± 3.37	71.86 ± 2.77	0.054
Chest width	64.57 ± 5.04	63.56 ± 3.09	66.61 ± 3.15	68.02 ± 3.19	0.067
Keel length	68.78 ± 3.44^B^	67.97 ± 2.55^B^	73.22 ± 2.43^A^	70.55 ± 2.90^AB^	0.002
Shank circumference	29.00 ± 1.20	28.67 ± 1.42	28.75 ± 2.20	27.46 ± 1.87	0.245
Shank length	39.19 ± 2.65	38.67 ± 1.28	40.25 ± 1.74	38.90 ± 1.41	0.301

^1^The results are expressed as mean ± SD (*n* = 90). ^AB^ Values within a row with no common superscripts differ significantly (*p* < 0.01).

**Table 6:** Different concentrations of *L. salivarius* S10 affect the 28-day slaughter performance of pigeons (g)^1^.

Items	CG	LG	MG	HG	*p* value
Dressed weight	403.6 ± 13.5^Bb^	407.5 ± 22.1^ABb^	432.9 ± 10.8^Aa^	421.1 ± 28.0^ABab^	0.014
Half-eviscerated weight	335.5 ± 15.1^Bb^	345.9 ± 26.4^ABb^	370.4 ± 11.5^Aa^	358.2 ± 36.1^ABab^	0.027
Eviscerated weight	298.1 ± 15.2^Bb^	307.6 ± 32.1^ABb^	333.2 ± 10.3^Aa^	320.2 ± 33.8^ABab^	0.031
Breast muscle weight	66.5 ± 7.9^Bb^	72.4 ± 7.7^ABab^	79.0 ± 4.3^Aa^	75.2 ± 7.9^ABa^	0.007

^1^The results are expressed as mean ± SD (*n* = 90). ^ab^Values within a row with no common superscripts differ significantly (*p* < 0.05). ^AB^Values within a row with no common superscripts differ significantly (*p* < 0.01).

### 3.5 *Ligilactobacillus salivarius* S10 alters intestinal morphology and gut microbiota in pigeons

**Figure 3:**
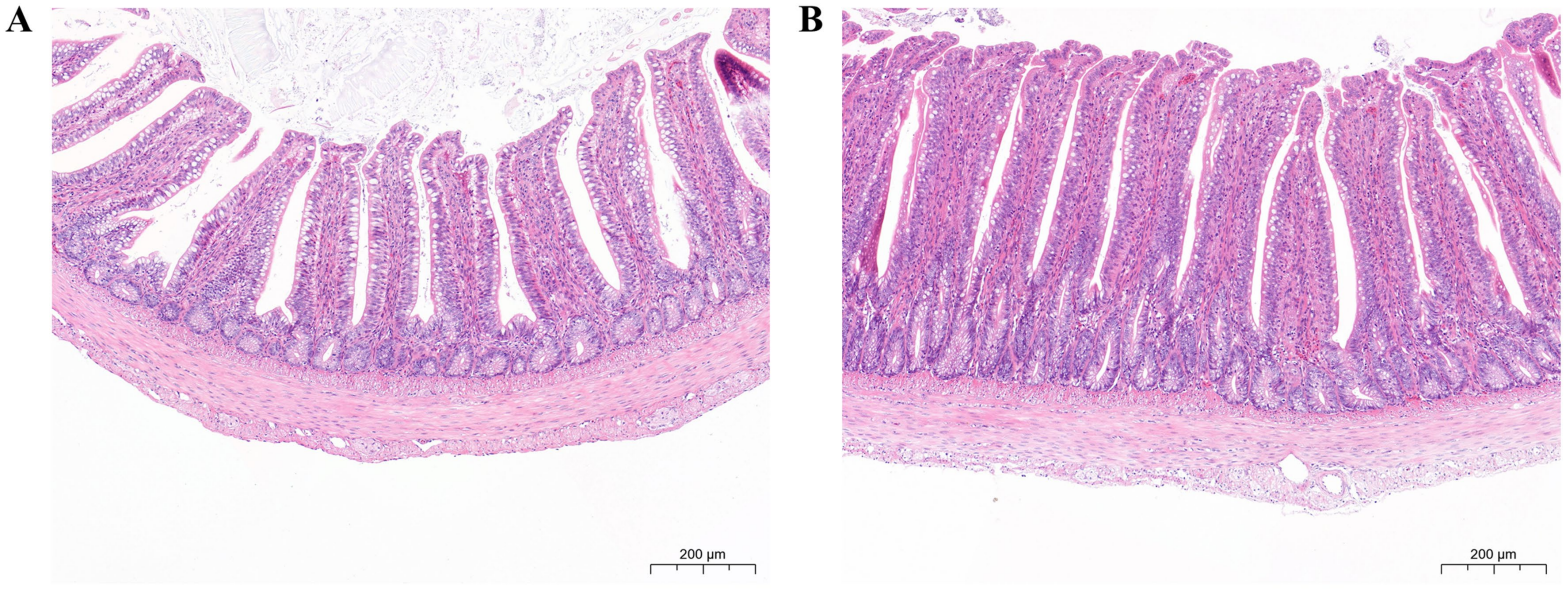
Ileum morphology by HE staining (14.0 ×). **(A)** Ileum morphology of CG. **(B)** Ileum morphology of MG. Scale bar: 200 μm.

In Figure 3, the ileum morphology of pigeons in CG and MG is shown under the microscope. After measurement, it was found that the villus height and the VH/CD of MG were significantly higher than those in CG (*p* < 0.01), but the crypt depth had no significant difference (*p* > 0.05) (Table 7). These findings suggest that the medium concentration of *L. salivarius* S10 may positively influence the intestinal morphology of newborn pigeons.

**Table 7:** Effect of *L. salivarius* S10 on the ileal morphology of pigeons^1^.

Items	CG	MG	*p* value
Villus height (μm)	407.6 ± 34.5^B^	482.8 ± 24.6^A^	< 0.001
Crypt depth (μm)	107.1 ± 14.0	111.6 ± 13.8	0.382
VH/CD	3.8 ± 0.5^B^	4.4 ± 0.5^A^	0.006

^1^The results are expressed as mean ± SD (*n* = 15). ^AB^Values within a row with no common superscripts differ significantly (*p* < 0.01).

**Figure 4:**
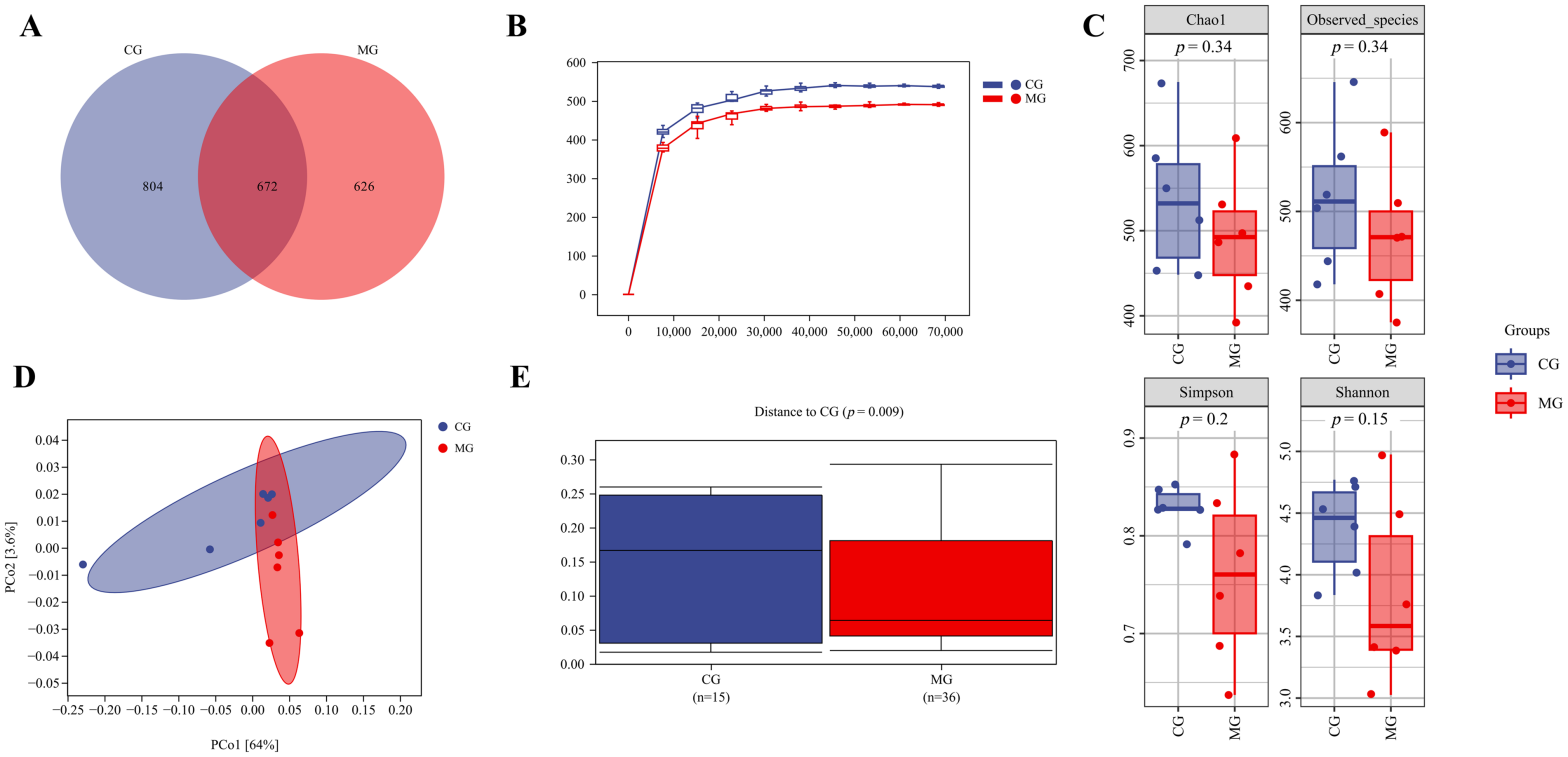
The diversity of the ileal microbiota. **(A)** Venn diagram. **(B)** Rarefaction curve. **(C)** Alpha diversity analysis. **(D)** PCoA based on Weighted UniFrac algorithm. **(E)** Sample distances based on Weighted UniFrac algorithm. Sample sizes for each group are as follows: CG (*n* = 6), MG (*n* = 6).

To further investigate the potential impact of *L. salivarius* S10 on the gut microbiota of these pigeons, we conducted alpha and beta diversity analysis. The collected ileal contents were submitted for gut microbiota analysis by 16S rRNA gene sequencing, and the sequencing depths results are depicted in Supplementary Table 1. The high-quality sequences in each sample (There were 12 samples in total and 6 samples in each group) were clustered into ASVs with 100% agreement, and the Venn diagram showed that total 1,476 and 1,298 ASVs were obtained from CG and MG, respectively, with 672 shared ASVs between two groups (Figure 4A). The rarefaction curve was constructed through counting the Chao1 index values of the samples. The curve tends to be flat, indicating that the sequencing depth of each sample is sufficient, and the sequencing results can be used to reflect the diversity contained in the sample (Figure 4B). No significant difference was found in a series of alpha diversity indices between CG and MG (Figure 4C). However, PCoA based on Weighted UniFrac distances algorithm revealed that the microbial communities in the ileal contents of the two groups were significantly different (*p* = 0.009) (Figures 4D–E).

### 3.6 Associations of differential ileal bacteria with production performance and intestinal development

**Figure 5:**
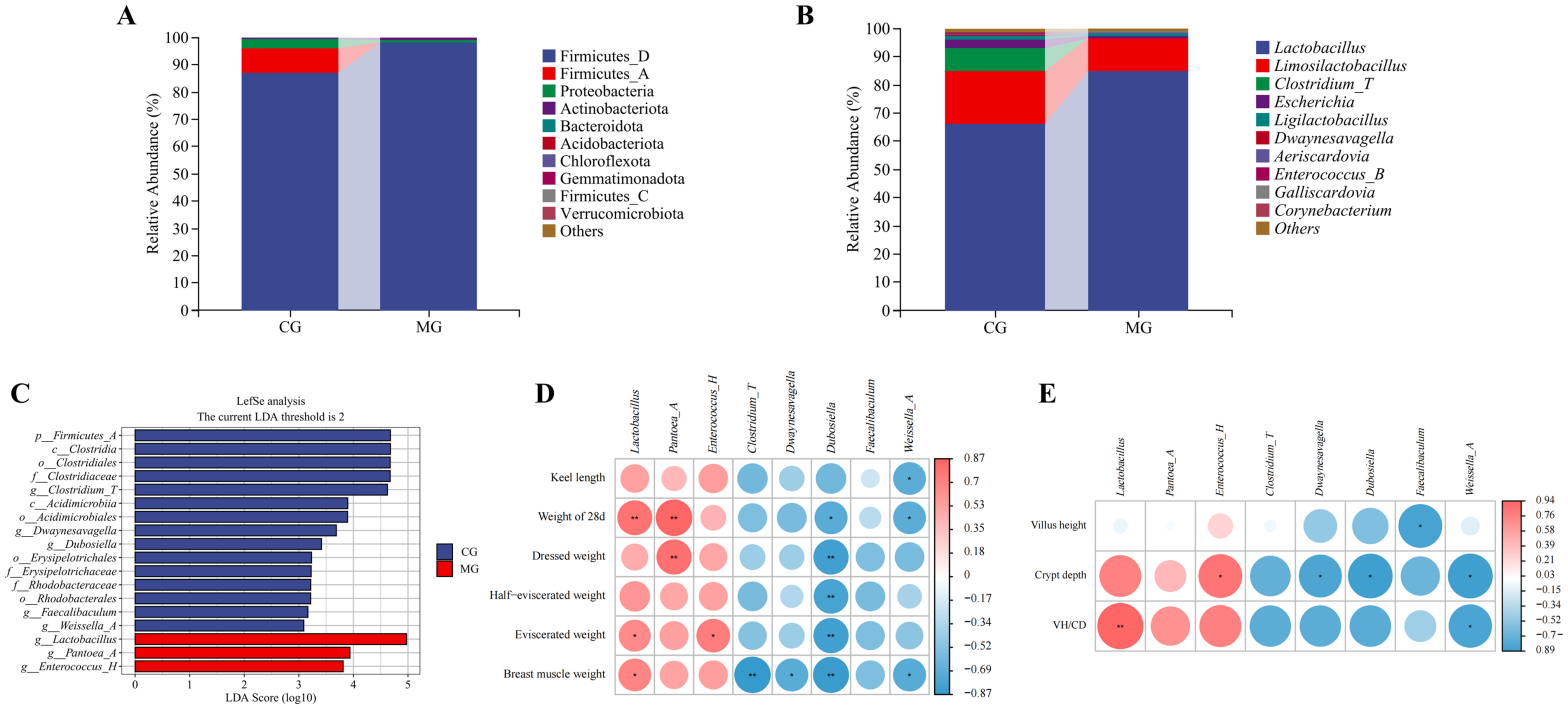
Differences in the ileal microbiota composition and associations of differential bacteria with production performance and intestinal development. **(A)** Bacterial community compositions at phylum level. **(B)** Bacterial community compositions at genus level. **(C)** LEfSe analysis. **(D)** Heatmap of the Spearman’s correlation analysis between differential ileal bacteria and production indexes of pigeons. The red and blue color represents a positive and negative correlation, respectively. *Indicates a difference at *p* < 0.05, **Indicates a difference at *p* < 0.01. **(E)** Heatmap of the Spearman’s correlation analysis between differential ileal bacteria and ileal morphological indexes of pigeons. The red and blue color represents a positive and negative correlation, respectively. *Indicates a difference at *p* < 0.05, **indicates a difference at *p* < 0.01. Sample sizes for each group are as follows: CG (*n* = 6), MG (*n* = 6).

To investigate the effects of *L. salivarius* S10 supplementation on intestinal flora, the relative abundance of two groups of samples at phyla and genus levels was analyzed. At the phylum level, Firmicutes (96.33 and 98.36%) was the dominant taxonomic unit in CG and MG, followed by Proteobacteria (3.16 and 0.91%), Actinobacteria (0.44 and 0.66%) and Bacteroidota (0.02 and 0.01%) (Figure 5A). At the genus level, the five dominant genera with the highest relative abundance in CG were *Lactobacillus* (66.35%), *Limosilactobacillus* (18.86%), *Clostridium_T* (8.06%), *Escherichia* (3.08%), and *Ligilactobacillus* (0.89%). In MG, the five dominant genera with the highest relative abundance were *Lactobacillus* (84.81%), *Limosilactobacillus* (11.75%), *Ligilactobacillus* (0.90%), *Escherichia* (0.82%), and *Aeriscardovia* (0.47%) (Figure 5B). Analysis with the LEfSe algorithm revealed that a total of 18 biomarkers with statistical differences (*p* < 0.05) were found after Wilcoxon test. Among these, 15 biomarkers were enriched in CG, which was characterized by *Clostridium_T* (LDA = 4.62), *Dwaynesavagella* (LDA = 3.69), *Dubosiella* (LDA = 3.42) et al. In contrast, 3 biomarkers were enriched in MG, such as *Pantoea_A* (LDA = 3.94), *Enterococcus_H* (LDA = 3.81). The highest LDA scores was given to *Lactobacillus*, which was considered as the most critical species in MG (LDA = 4.97) (Figure 5C). The relative abundance of *Lactobacillus* was significantly positively correlated (*p* < 0.05) with eviscerated weight and breast muscle weight, and highly significantly positively correlated (*p* < 0.01) with weight of 28 d and VH/CD. The relative abundance of *Pantoea_A* was highly significantly positively correlated (*p* < 0.01) with weight of 28 d and dressed weight. The relative abundance of *Enterococcus_H* was significantly positively correlated (*p* < 0.05) with eviscerated weight and crypt depth. In contrast, biomarkers in CG showed largely negative regulatory relationships with production performance and intestinal development. The relative abundance of *Clostridium_T* showed a highly significant negative correlation with breast muscle weight (*p* < 0.01). The relative abundance of *Dwaynesavagella* showed a significant negative correlation (*p* < 0.05) with breast muscle weight and crypt depth. The relative abundance of *Dubosiella* was significantly negatively correlated with weight of 28 d and crypt depth (*p* < 0.05), and was highly significantly negatively correlated with dressed weight, half-eviscerated weight, eviscerated weight, and breast muscle weight (*p* < 0.01). The relative abundance of *Faecalibaculum* was significantly negatively correlated with villus height (*p* < 0.05). The relative abundance of *Weissella_A* was significantly negatively correlated with keel length, weight of 28 d, breast muscle weight, crypt depth, and VH/CD (*p* < 0.05) (Figures 5D,E).

### 3.7 Ileum transcriptome changes in pigeons after *Ligilactobacillus salivarius* S10 link MAPK pathway and gut microbiota

**Figure 6:**
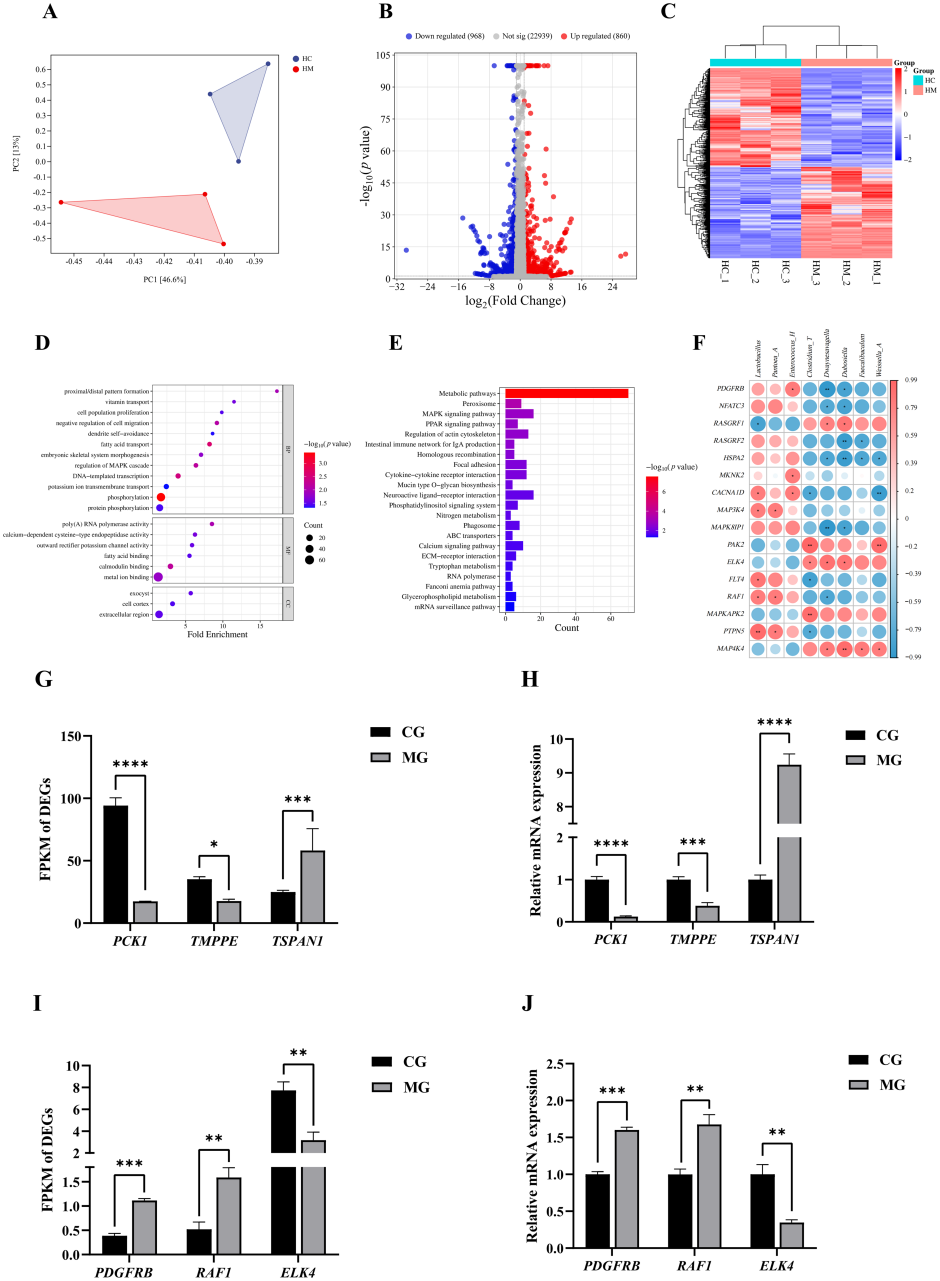
Ileal transcriptomics. **(A)** PCOA diagram between two groups of samples. **(B)** Volcano plot of DEGs between two groups. **(C)** Cluster heatmap. **(D)** Dot plot of the GO enrichment analysis. **(E)** Histogram of KEGG enrichment analysis. **(F)** Heatmap of the Spearman’s correlation analysis between differential ileal bacteria and DEGs in the MAPK signaling pathway. The red and blue color represents a positive and negative correlation, respectively. *Indicates a difference at *p* < 0.05, **Indicates a difference at *p* < 0.01. **(G)** FPKM values of DEGs randomly selected from outside the MAPK signaling pathway. *Indicates a difference at *p* < 0.05, **indicates a difference at *p* < 0.01, ***indicates a difference at *p* < 0.001, ****indicates a difference at *p* < 0.0001, same below. **(H)** Relative mRNA expression levels of DEGs randomly selected from outside the MAPK signaling pathway. **(I)** FPKM values of DEGs randomly selected from the MAPK signaling pathway. **(J)** Relative mRNA expression levels of DEGs randomly selected from the MAPK signaling pathway. Sample sizes for each group are as follows: CG (*n* = 3), MG (*n* = 3).

In order to get a comprehensive picture of the transcription of ileal genes in pigeons after gavage of *L. salivarius* S10, RNA-seq was performed in this study. All the statistical results after quality control of transcriptome sequencing data show that this data can be used for subsequent bioinformatics analysis, as detailed in Supplementary Table 2. PCA results indicated differences in intestinal transcription between CG and MG (Figure 6A). Therefore, we next calculated the DEGs between the two groups, and volcano plot was drawn. The results showed that when Fold change > 2 and *p* < 0.05 were set as the threshold for screening significant DEGs, a total of 1828 DEGs were noted in the ileum tissues of pigeons in CG and MG. Compared with CG, 860 genes were up-regulated and 968 genes were down-regulated in MG (Figure 6B). Meanwhile, the up-regulated and down-regulated genes were clearly distinguished among the groups, and the correlation among the samples in each group performs well (Figure 6C).

GO and KEGG enrichment analyses were conducted on 1828 DEGs to explore their functions. The GO analysis revealed 12 biological processes terms related to processes like MAPK cascade regulation, cell proliferation, and phosphorylation; 6 molecular functions terms associated with poly(A) RNA polymerase and calcium-dependent cysteine-type endopeptidase activities; and 3 cell components terms encompassing cell cortex, exocyst, and extracellular region (Figure 6D). These DEGs were mainly enriched in biological pathways such as Metabolic pathways, Peroxisome, MAPK signaling pathway, Regulation of actin cytoskeleton, Cytokine-cytokine receptor interaction (Figure 6E).

Notably, the MAPK signaling pathway, which was significantly enriched, plays a key role in animal growth and development, and gene expression of the MAPK signaling pathway may be affected by intestinal microbiota. Therefore, in the present study, the ileal differential bacteria were subjected to a Spearman’s correlation analysis with the genes involved in this pathway. For instance, the expression of *PDGFRB* showed a significant positive correlation with the relative abundance of *Enterococcus_H* and a significant negative correlation with the relative abundance of *Dubosiella* (*p* < 0.05). The expression of *RAF1* exhibited a significant positive correlation with the relative abundances of *Lactobacillus* and *Pantoea_A*, while demonstrating a notable negative correlation with the relative abundance of *Dwaynesavagella* (*p* < 0.05). The expression level of *HSPA2* was significantly negatively correlated with the relative abundance of *Dwaynesavagella*, *Faecalibaculum*, and *Weissella_A* (*p* < 0.05). All sixteen differentially expressed genes found to correlate with ileal differential bacteria were in Figure 6F. Finally, we randomly selected *PDGFRB*, *RAF1*, and *ELK4* from within the MAPK pathway, and *PCK1*, *TMPPE*, and *TSPAN1* from outside this pathway, for further validation using qRT-PCR. The results from qRT-PCR showed a high degree of agreement with the data from the transcriptome analysis, reinforcing the robustness and credibility of our RNA-Seq data (Figures 6G–J).

## 4 Discussion

LAB, ubiquitous in diverse ecological habitats ranging from fermented or spoiled food to environmental matrices and animal guts, play a pivotal role in maintaining intestinal flora balance and are prime probiotic candidates (Duar et al., 2017b; Zhang et al., 2018). As gram-positive, facultative anaerobes, LAB thrive in weak acid environments and require vitamins, amino acids, and peptides for growth (Mokoena, 2017). In this study, we isolated 20 strains of *L. salivarius* from the intestines of healthy pigeons using MRS medium. These strains exhibited probiotic potential, as previously reported in chickens (Jiang et al., 2023), cows (Lin et al., 2020), piglets (Sun et al., 2020), and bees (Fanciotti et al., 2018). Among these strains, S5, S7, S10, S11, and S19 demonstrated potent antibacterial activity against crucial livestock pathogens, including *E. coli*, *S. aureus*, and *Salmonella*. Their rapid proliferation and lactic acid production further underscore their probiotic efficacy, evidenced by a reduction in pH in bacterial solutions.

Adapting to the gastrointestinal environment is crucial for LAB with robust bacteriostatic ability or fertility to exert their biological effects (Luo et al., 2022). Therefore, we evaluated the adaptability of these strains through *in vitro* tests by altering medium conditions. All strains exhibited resistance, aligning with findings on *L. salivarius* CML352 (Xu et al., 2022) and *L. salivarius* SMXD51 (Messaoudi et al., 2011). Notably, Despite its slight disadvantage in terms of acid and enzyme resistance, S10 is undoubtedly the most suitable strain for pigeon preparation due to its superior overall antimicrobial capacity and its resistance to artificially simulated gastrointestinal fluids compared to other strains. This strain’s adaptability suggests a potential for practical application, particularly in the context of host-specific microbiota development. The host-specific microbiota development benefits both the host and the species (Yuan et al., 2022). By fostering a beneficial microbial environment tailored to the host, we can observe positive outcomes in growth and overall health. For instance, feeding chicks T-Pbx, a probiotic blend derived from turkey ileum, improved weight gain (Ward et al., 2019). Similarly, direct dosing of newborn pigeons with *L. salivarius* S10 maximized benefits, enhancing the performance of 28-day-old pigeons by improving keel length, weight, and breast muscle weight. This underscores the potential of *L. salivarius* S10 as a probiotic for pigeons, reinforcing the idea that strain selection based on adaptability can lead to significant improvements in host health and performance.

LAB’s glucose metabolism produces organic acids, facilitating mineral absorption and nutrient utilization (Scholz-Ahrens et al., 2007). During evolution, pigeons have adapted their intestinal structure to facilitate flight, with the large intestine degenerating to primarily absorb water, while the small intestine handles most digestion and nutrient absorption (Chen et al., 2015). The ileum, as the terminal site for absorbing carbohydrates, proteins, and fats, hosts a complex microbiota closely linked to pigeon health and development. Additionally, it serves as a key site for evaluating whether screened probiotic strains can survive and colonize the post-digestive segment. Given these characteristics, future experiments will focus on ileal development and microbiota structure. In our study, increased villus height indicated enhanced digestion and absorption, positively impacting growth (Zhu et al., 2022). Additionally, we observed changes in the intestinal microbial communities of the pigeons. Specifically, there was an increase in Firmicutes, represented by *Lactobacillus*, and a decrease in Proteobacteria. Similarly, probiotics have been shown to alter gut microbiota in mice (Park et al., 2013). In pigeons, the abundance of *Lactobacillus*, *Pantoea_A*, and *Enterococcus_H* rose, while *Limosilactobacillus*, *Clostridium_T*, and *Escherichia* decreased. The abundance of *Ligilactobacillus* remained stable, possibly due to interference competition (Ghoul and Mitri, 2016). These findings highlight the ability of *L. salivarius* S10 to modulate the gut microbiota of pigeons favorably, contributing to their overall health and growth performance.

In exploring the intricate relationships between gut microbiota and growth performance in pigeons, we conducted a detailed analysis of microbial community shifts following probiotic administration. Spearman’s correlation analysis linked *Lactobacillus* abundance to positive growth traits, while *Dubosiella* and *Faecalibaculum* were negatively correlated with intestinal health indicators (Gu et al., 2023; Ran et al., 2024). In addition to *Lactobacillus*, a common probiotic, strains with probiotic potential also exist in *Enterococcus_H*. Studies have shown that the addition of *Enterococcus faecium* improves the intestinal microecology of broilers and increases their average weight gain, and also helps to lower cholesterol levels in hypercholesterolemic mice (Zhu et al., 2019; Suvorov et al., 2023). *Pantoea ananatis* from *Pantoea_A* has been used to produce L-cystine feed additives (Bampidis et al., 2020). The lipopolysaccharide IP-PA1 extracted from *Pantoea agglomerans* is able to maintain homeostasis in the body *via* macrophage activation and has immune functions such as healing, analgesia, and protection against infections, allergies, and cancer (Nakata et al., 2011). On the contrary, *Clostridium perfringens*, which was significantly enriched in *Clostridium_T* in CG, caused necrotizing enteritis and biliary hepatitis in poultry (Van Immerseel et al., 2004).

These correlations provide insights into how specific microbial species may influence the overall health and development of the host. Notably, early niche pre-emption led to *Lactobacillus* dominance in the intestines of 28-day-old pigeons, reducing microbiota diversity—a potential sign of dysbiosis. This observation underscores gut microbial colonization in suckling pigeons during development to 28 days of age and the need for careful modulation to maintain a balanced microbial ecosystem. However, our findings indicate that *L. salivarius* S10 regulated the gut flora to a growth-favorable state, potentially beneficial for short-lived animals like pigeons. This may be attributed to the fact that *L. salivarius* can regulate the composition of the gut microbiota through the production of lactic acid, H₂O₂, bacteriocins, short-chain fatty acids (SCFAs), and others (Yang et al., 2024). These active metabolites work by fostering an environment conducive to *Lactobacillus* dominance while mitigating potential negative impacts of reduced diversity. It is evident that *L. salivarius* S10 emerges as a promising probiotic for enhancing the growth and health of pigeons. However, due to the relatively small number of pigeons in this study, the findings may not be broadly generalizable, and a larger sample size is needed to accurately validate the results. Meanwhile, future research should determine whether long-term supplementation with *L. salivarius* S10 can maintain the long-term balance and stability of the intestinal microbiota. Additionally, a comprehensive safety assessment of *L. salivarius* S10 is necessary to identify any potential risks and ensure its suitability for commercial use.

To further elucidate the mechanisms underlying these observations, we conducted RNA-seq analysis of the ileum, which revealed 1828 differentially expressed genes (DEGs). Among these DEGs, several were enriched in the MAPK (mitogen-activated protein kinase) signaling pathway, a critical pathway for growth and development. This enrichment provides a biological context for understanding how gut microbiota may influence host growth. Notably, *RAF1*, a core member of the MAPK pathway, was found to be correlated with differential bacteria. *RAF1* is a key kinase that initiates the MAPK cascade in response to extracellular stimuli, ultimately regulating cell proliferation, differentiation, and survival. Our findings suggest that specific gut bacteria may interact with *RAF1* or its upstream regulators, thereby modulating the MAPK pathway and influencing host growth traits. In addition to *RAF1*, *PDGFRB* also emerged as a key player in gut development. Studies have shown that PDGF signaling is involved in the proliferation and differentiation of intestinal epithelial cells (Sukhotnik et al., 2012; Wang et al., 2019), and upregulation of this gene also contributes to the observed changes in intestinal morphology and nutrient absorption. And with further research, there have been reports demonstrating the role of *L. salivarius* metabolites in regulating host signaling pathways. SCFAs such as butyric acid can promote the proliferation and repair of intestinal epithelial cells through activation of ERK and p38 MAPK pathways (Mann et al., 2024). Succinic acid promotes the activity of intestinal stem cells by activating the SUCNR1-mitochondrial axis, thereby enhancing the renewal and absorption of intestinal epithelial cells (Luo et al., 2025). Therefore, we hypothesized that the intestinal tract’s ability to digest and absorb nutrients was enhanced, which in turn improved the production performance of suckling pigeons.

This study highlights the potential of *L. salivarius* S10 as a probiotic for pigeons, demonstrating its ability to enhance growth performance and regulate gut flora. The RNA-seq analysis provides valuable insights into the mechanisms underlying these effects, specifically suggesting that intestinal microbiota regulate MAPK pathway genes, thereby influencing intestinal development and nutrient utilization. These findings further elaborate on the complex interplay between gut microbiota and host signaling pathways, with profound implications for the growth and health of pigeons. Our results not only deepen our understanding of the microbial-host interactions that govern avian physiology but also open up new avenues for the development of probiotics and other microbial-based interventions tailored to enhance pigeon farming productivity. By harnessing the power of the gut microbiome through *L. salivarius* S10 supplementation, we can potentially optimize growth rates, improve feed conversion efficiency, and bolster disease resistance in pigeons, ultimately contributing to more sustainable and profitable poultry operations. Thus, the ongoing exploration of the gut microbiota’s regulatory role in host signaling pathways, particularly as facilitated by probiotics like *L. salivarius* S10, holds immense promise for revolutionizing the future of pigeon agriculture. Future studies could explore these genes at the cellular level to further elucidate their roles in intestinal health and growth promotion, thereby building upon the foundation laid by this research and advancing our understanding of probiotics in pigeons.

## 5 Conclusion

In this study, we successfully isolated a strain of *L. salivarius* S10, known for its exceptional probiotic properties, from the ileal contents of 28-day-old Mimas pigeons. Our findings demonstrated that the administration of this strain to young pigeons led to a notable enhancement in their overall production performance. Specifically, under the experimental conditions employed, the optimal dosage of *L. salivarius* S10 was determined to be 10^9^ CFU/mL. This concentration not only resulted in improvements in the intestinal histomorphology of the pigeons but also induced significant alterations in their ileal intestinal flora and transcriptome profiles when compared to the control group (CG). These results underscore the potential of *L. salivarius* S10 as an effective probiotic for pigeons, highlighting its ability to positively impact gut health and overall growth performance. Future studies should focus on evaluating the long-term effects of *L. salivarius* S10 supplementation on intestinal microbiota stability, exploring its potential effects, and assessing its safety profile in larger-scale trials to ensure its suitability for commercial application in pigeon farming.

## Data Availability

The data supporting this article are available in the supplementary information. The sequences of the 20 isolated strains have been deposited in the NCBI database (https://www.ncbi.nlm.nih.gov/) under accession numbers PP813760-PP813779. High-throughput sequencing datasets have also been deposited in the NCBI database under accession numbers PRJNA1112620 and PRJNA1114431.

## Footnotes

^1^ https://www.ncbi.nlm.nih.gov/

^2^ https://www.megasoftware.net/

^3^Release 13.8, https://www.genescloud.cn/home.

^4^ https://david.ncifcrf.gov/

^5^ http://bioinfo.org/kobas

^6^ https://www.bioinformatics.com.cn

^7^ https://www.genescloud.cn/login
