# Supplementary material for: Probiotic efficacy and mechanism of a pigeon derived Ligilactobacillus salivarius strain in promoting growth and intestinal development of pigeons
Source: Front Microbiol. 2025 May 9;16:1584380. doi: 10.3389/fmicb.2025.1584380 (PMC12098536; doi:10.3389/fmicb.2025.1584380)
Supplement: Supplementary file 1 [file Data_Sheet_1.pdf]

Supplementary Material

Supplementary Table 1. Sequencing depth of ileal microbiota of pigeons in CG and MG.

SampleID	Input[1]	Filtered[2]	Denoised3[3]	Merged[4]	Non-chimeric[5]	Non-singleton[6]
C1	109087	102699	101864	100846	80340	80321
C2	114350	108308	107684	106784	94571	94551
C3	115709	109304	108466	106717	92184	92162
C4	114712	109083	108274	107816	93456	93437
C5	117841	110908	109396	106198	76470	76419
C6	115153	108717	107926	106374	91207	91188
M1	110759	103424	102823	102295	91701	91698
M2	97732	90914	90267	89267	77896	77885
M3	103606	96262	95598	94000	63900	63882
M4	118489	110270	109747	109142	82488	82476
M5	107282	100327	99665	98850	75926	75915
M6	107390	99800	99228	98832	85881	85879

[1] The sequence quantity of both forward and reverse primers can be matched in the original data. [2] The sequence quantity after removing low quality sequence. [3] The sequence quantity after denoising. [4] The sequence quantity after concatenation. [5] The sequence quantity after filtering chimeras. [6] The sequence quantity after filtering singletons.

Supplementary Table 2. Quality control and comparison of transcriptome sequencing in pigeon ileum.

Sample ID[1]	Raw reads[2]	Clean reads[3]	Q20(%)[4]	Q30(%)[5]	Mapping rate(%)[6]
HC_1	52694802	52195650	97.07%	92.14%	76.30%
HC_2	49258364	48768966	96.83%	91.64%	74.95%
HC_3	47619980	47248032	97.23%	92.49%	77.87%
HM_1	49415182	49006350	97.15%	92.32%	76.64%
HM_2	53380322	52909070	97.18%	92.42%	76.44%
HM_3	59027310	58567376	97.43%	92.96%	76.50%

[1] HC represents the ileum samples of CG and HM represents the ileum samples of MG. [2] Number of reads after original sequencing. [3] Number of filtered reads. [4] The number of bases with phred quality score greater than or equal to 20 as a percentage of the total number of bases. [5] The number of bases with phred quality score greater than or equal to 30 as a percentage of the total number of bases. [6] Reads that could be compared to reference genomes accounted for all Clean Reads.

S1

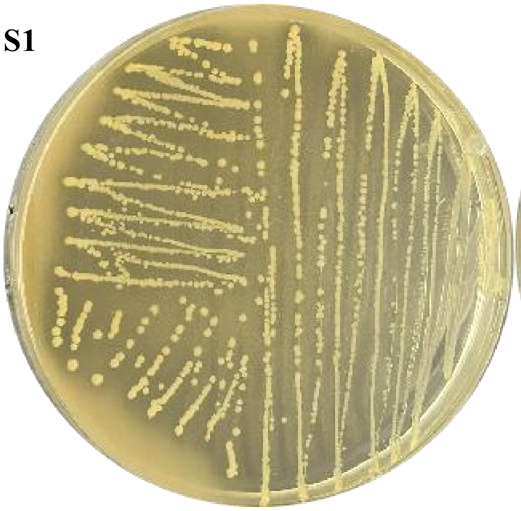

S2

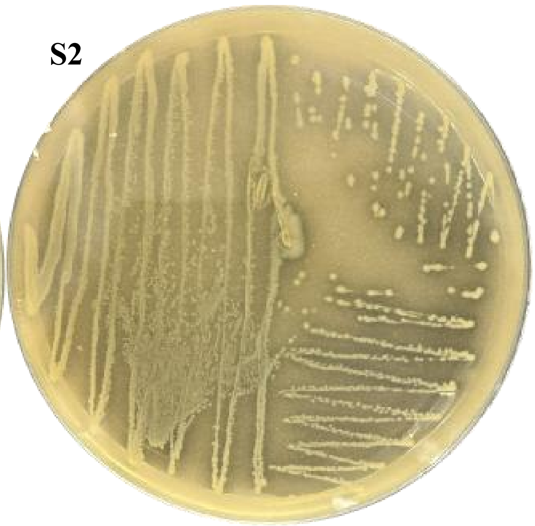

S3

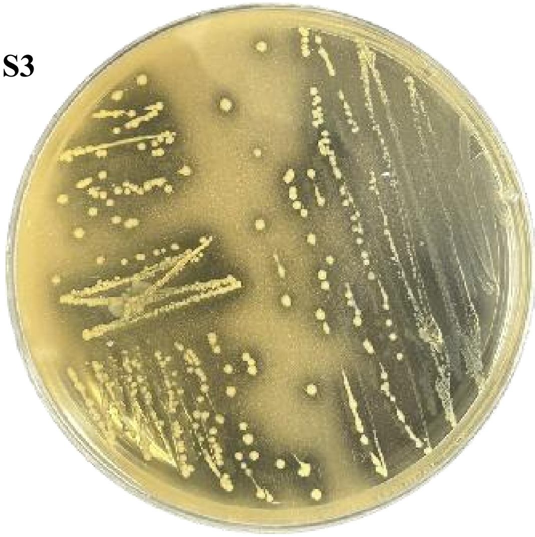

S4

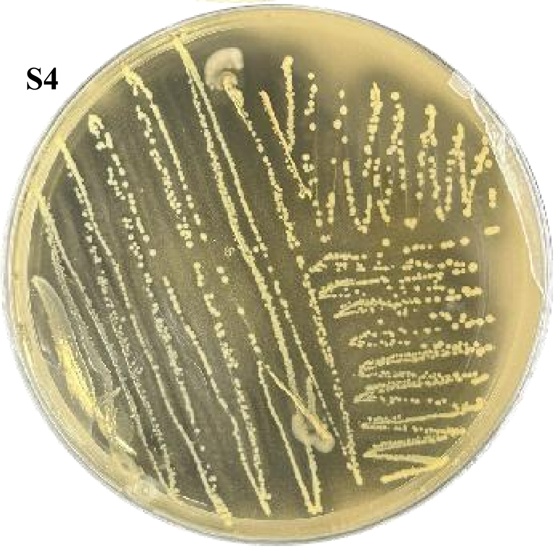

S5

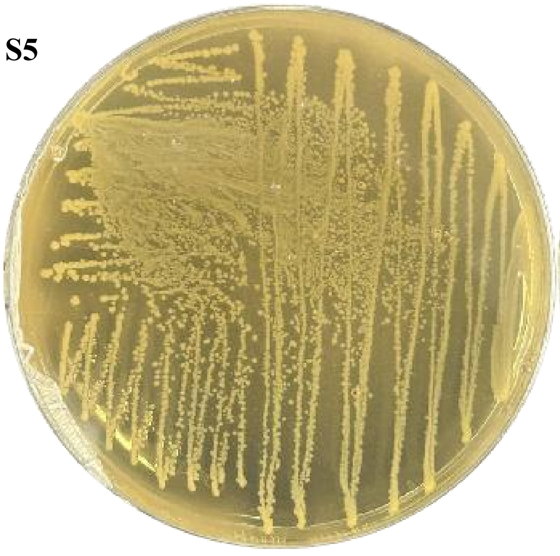

S6

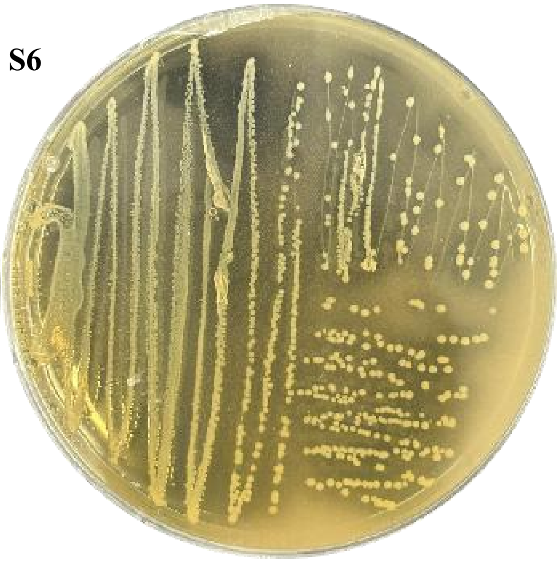

S7

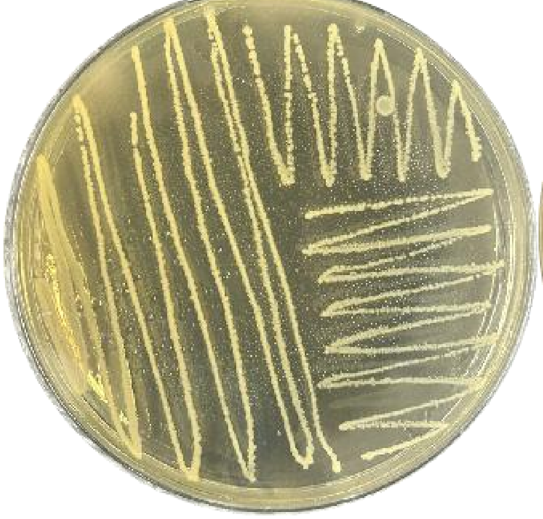

S8

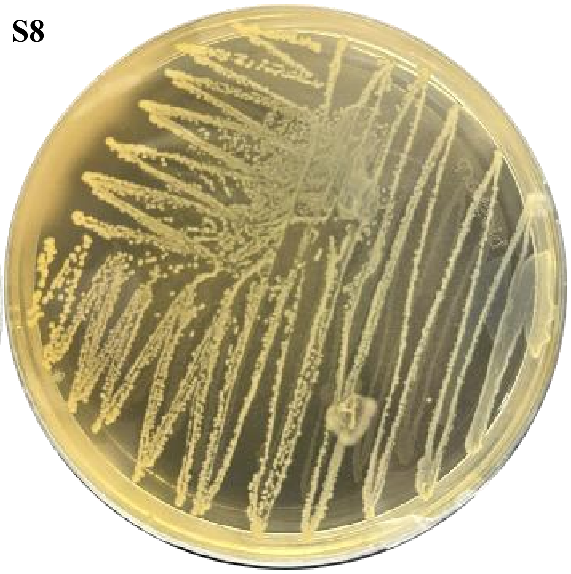

S9

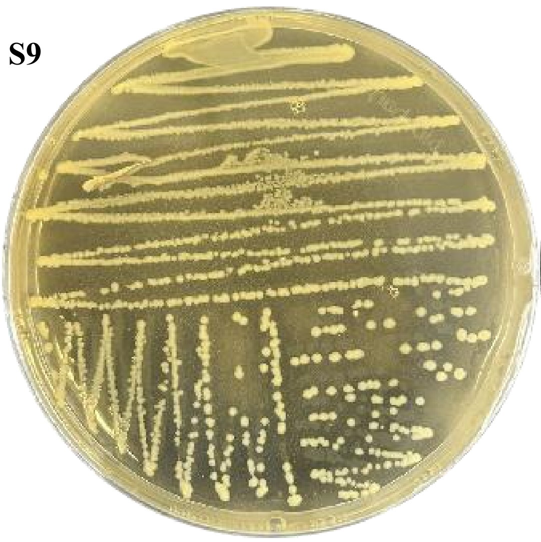

S10

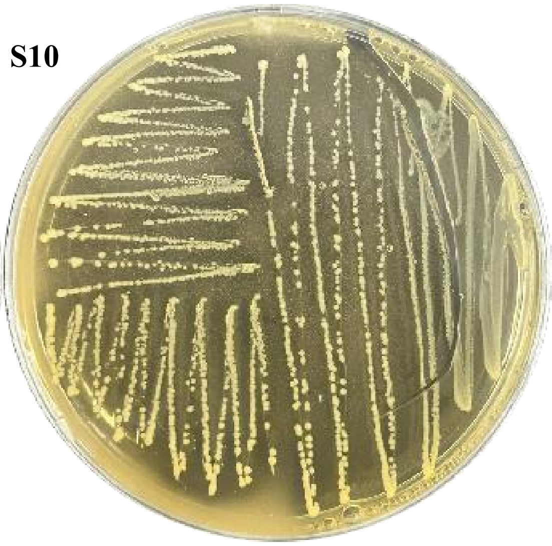

S11

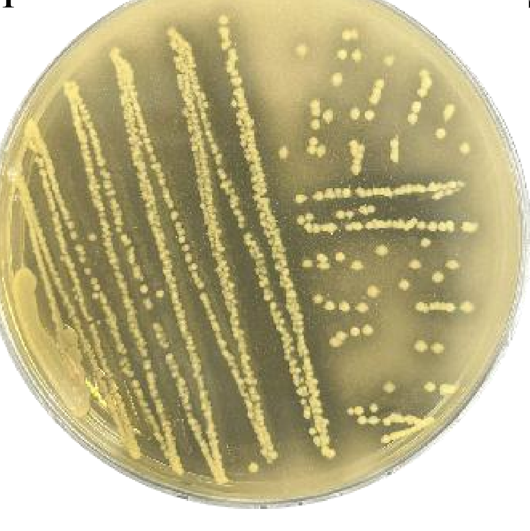

S12

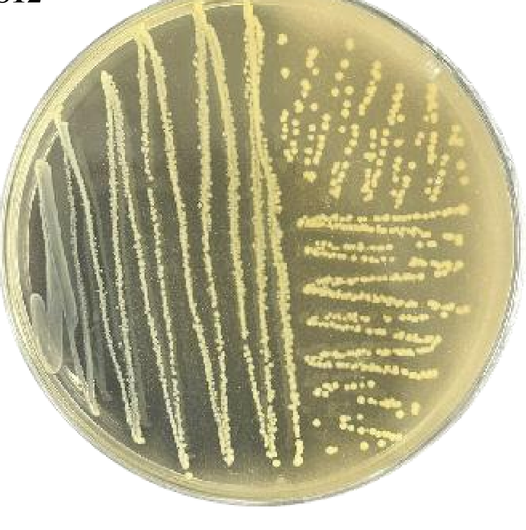

S13

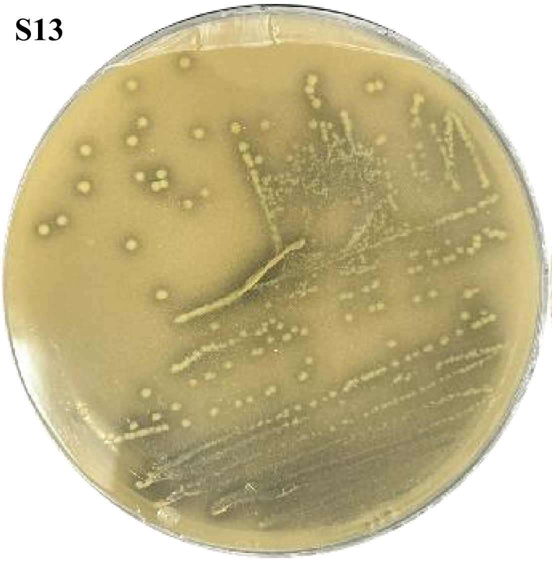

S14

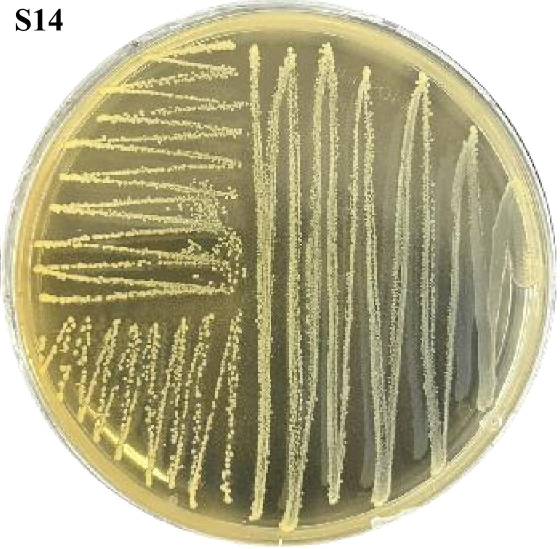

S15

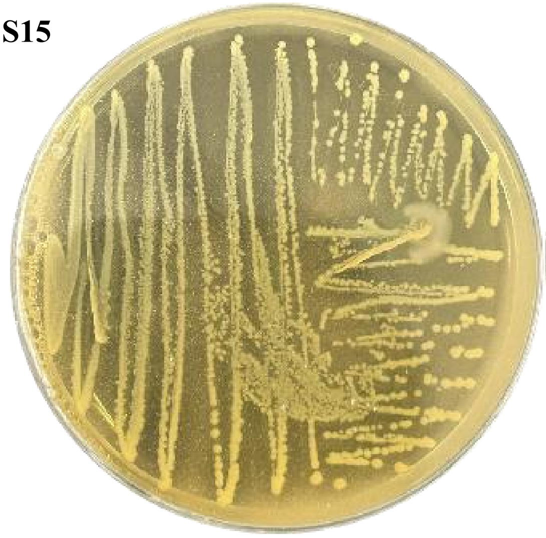

S16

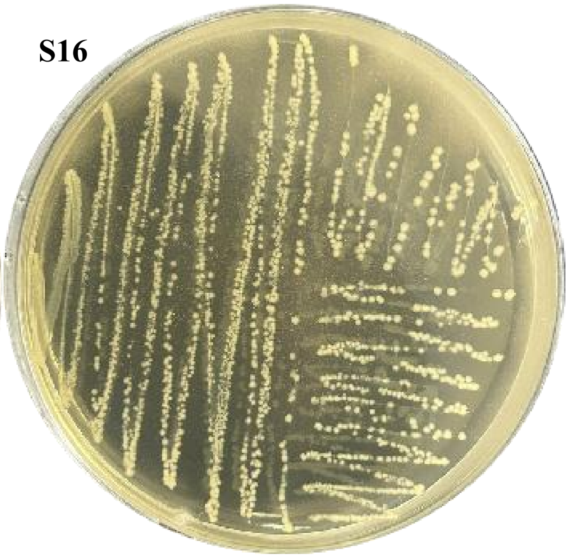

S17

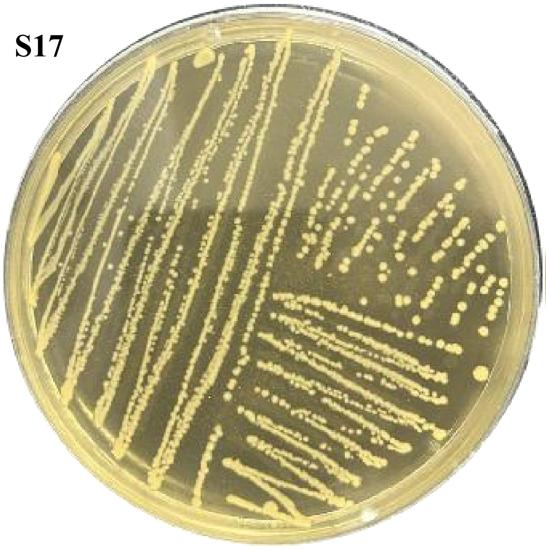

S18

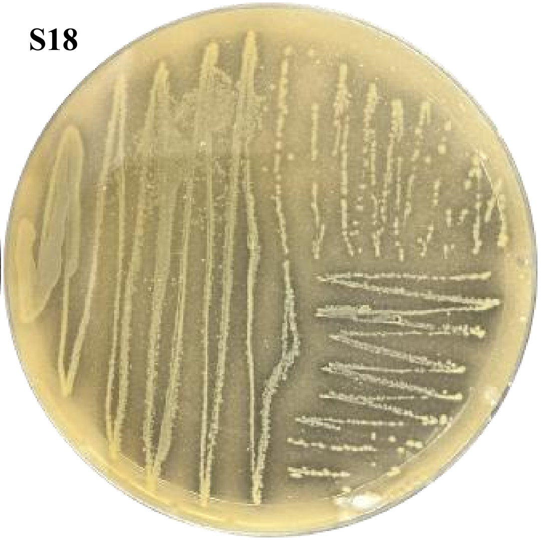

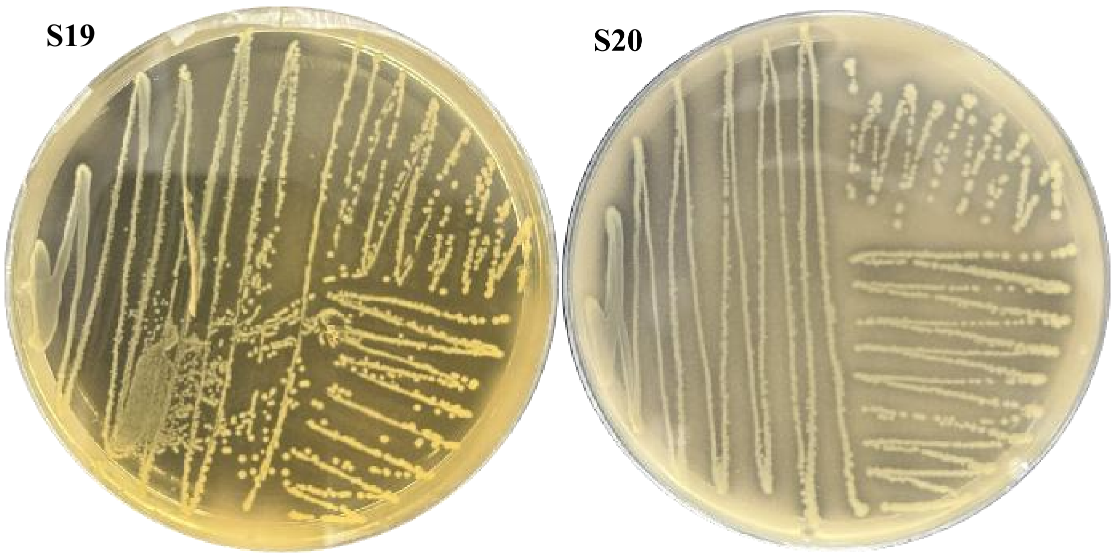

Supplementary Figure 1. Colony characteristics of 20 isolated strains.

S1

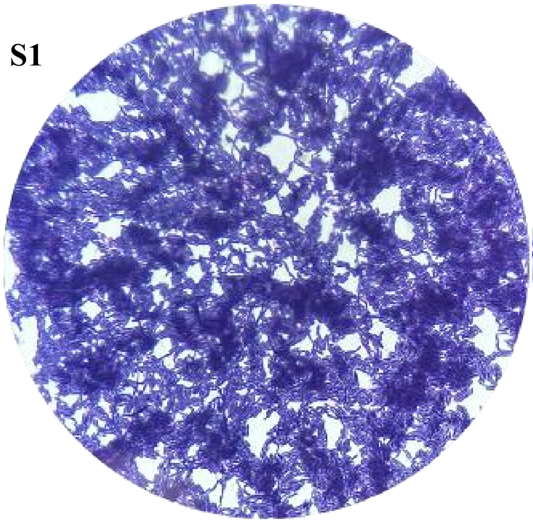

S2

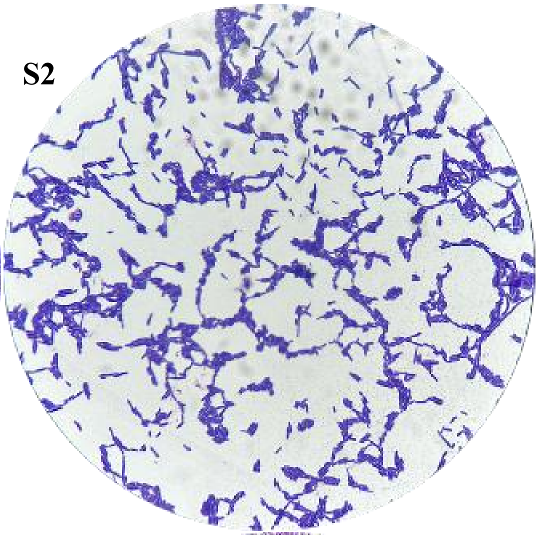

S3

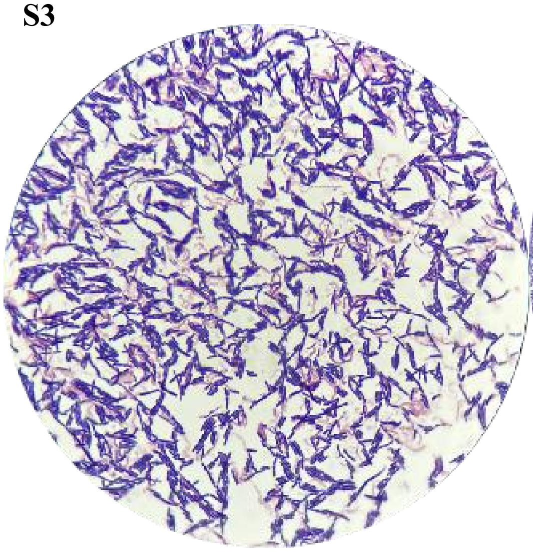

S4

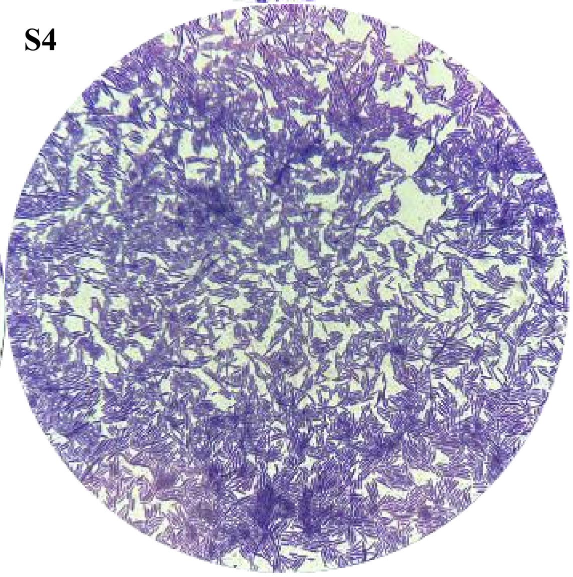

S5

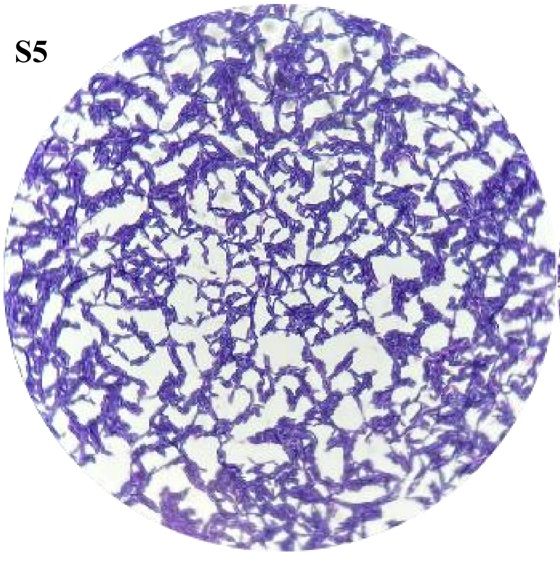

S6

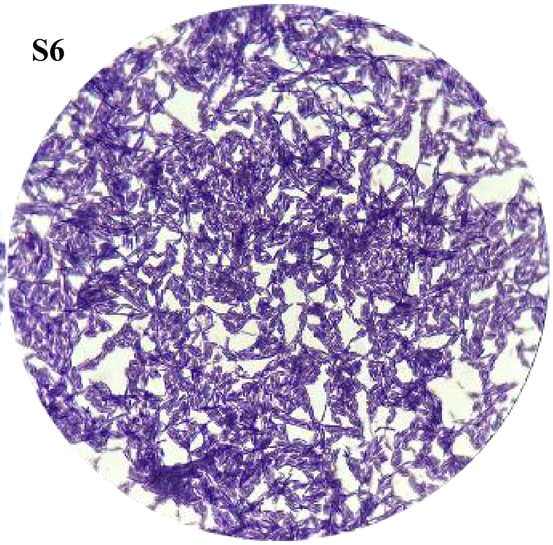

S7

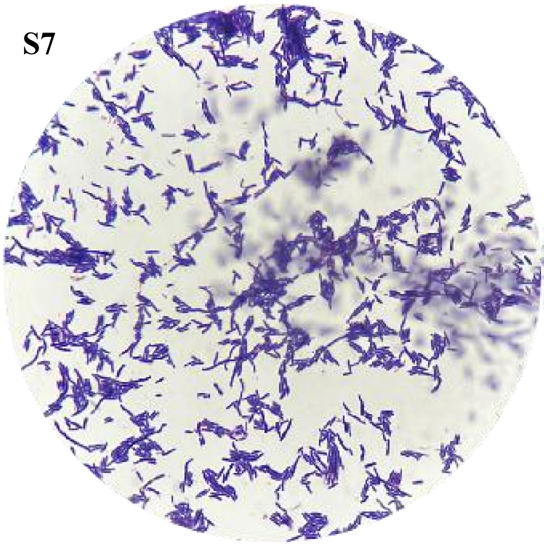

S8

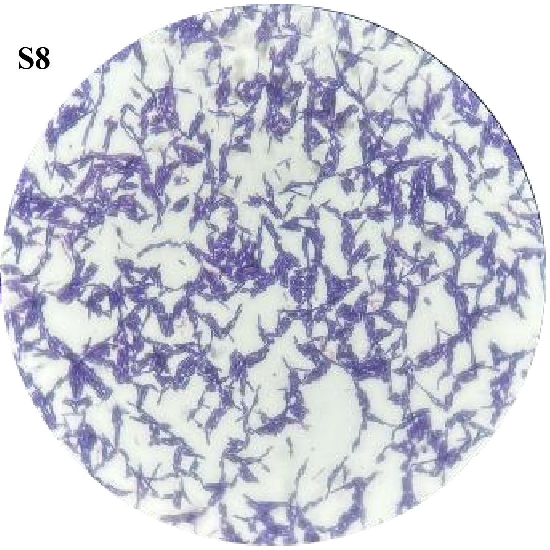

S9

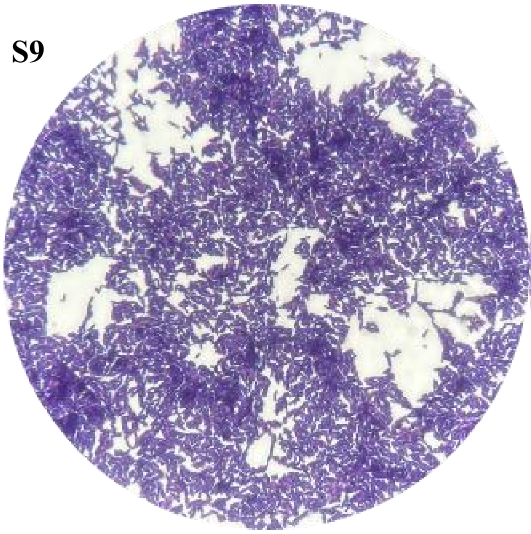

S10

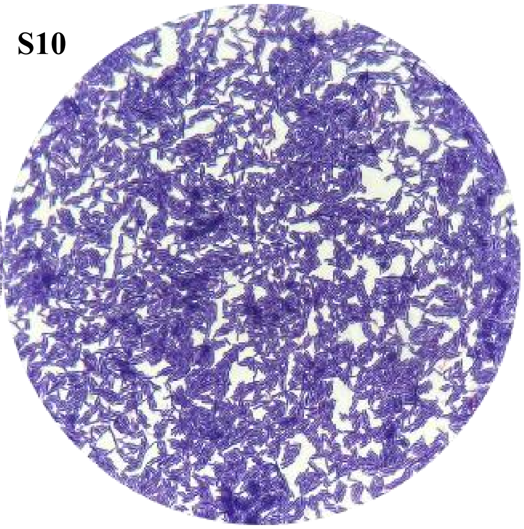

S11

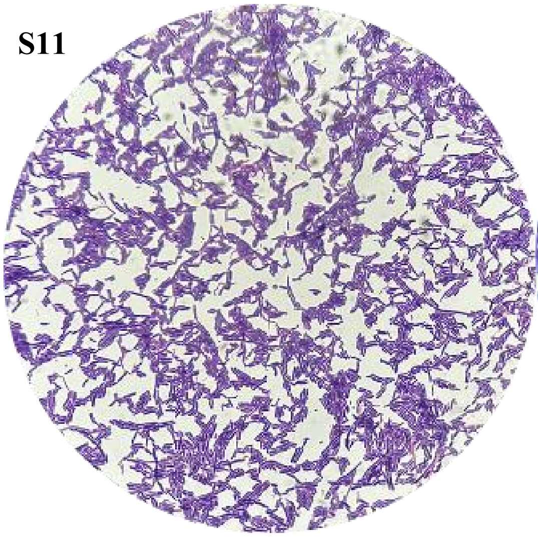

S12

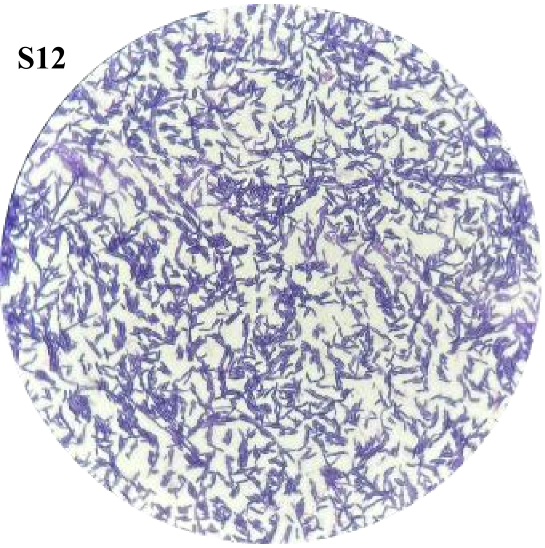

S13

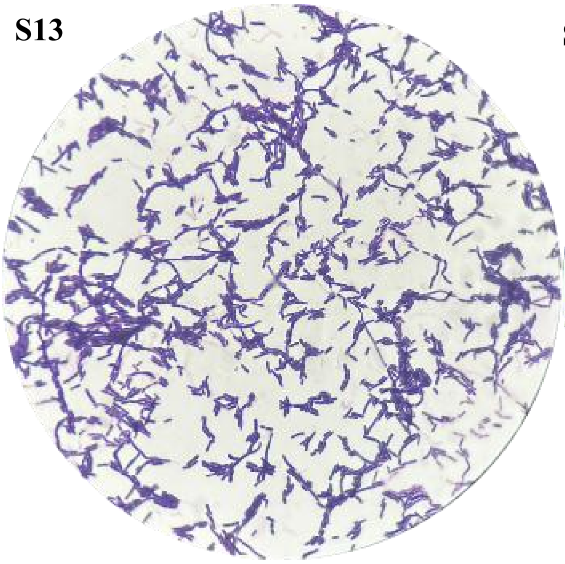

S14

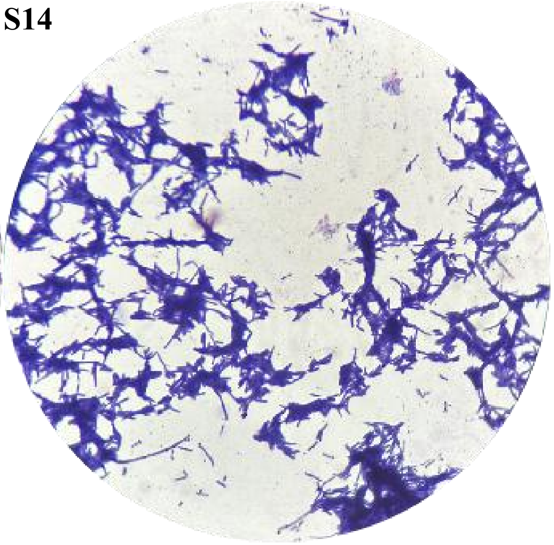

S15

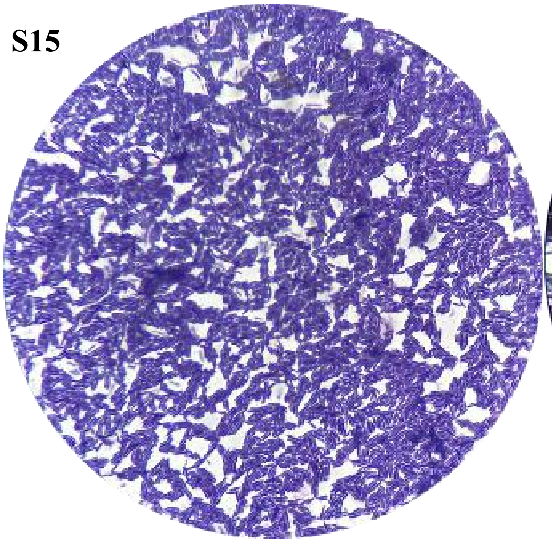

S16

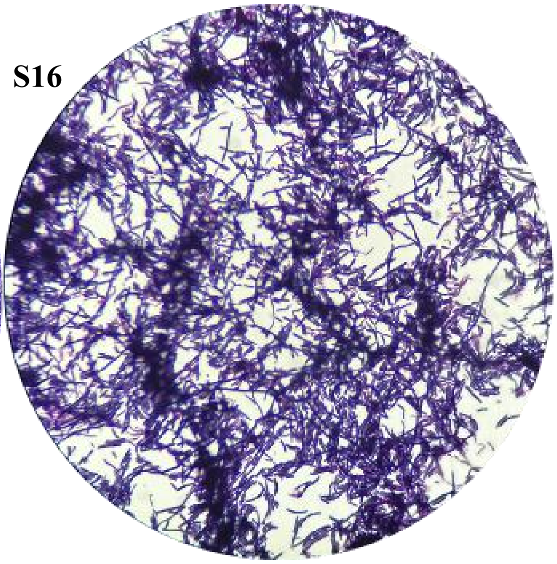

S17

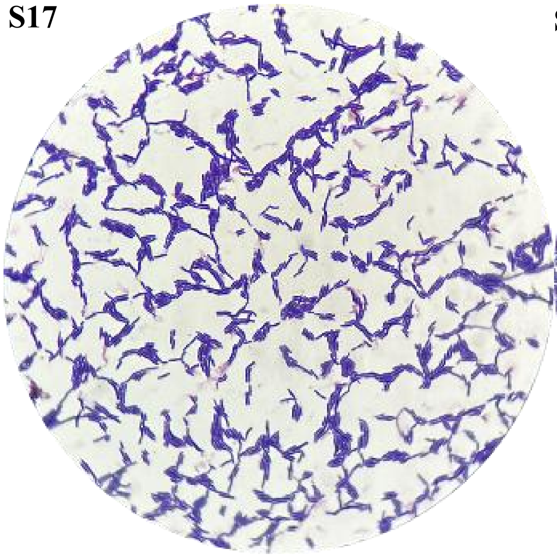

S18

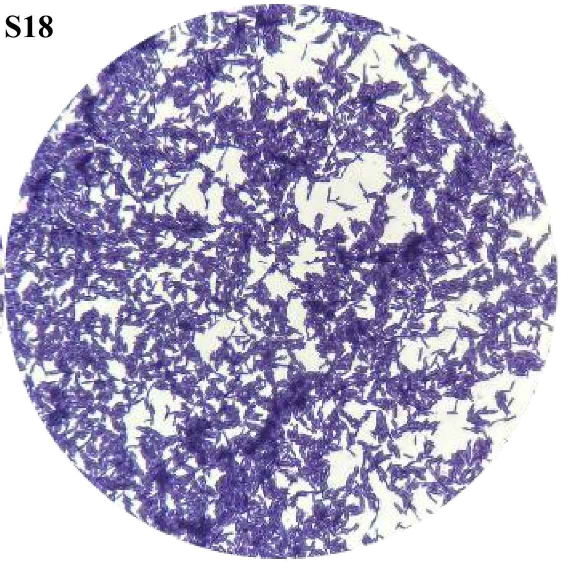

S19 **S20**

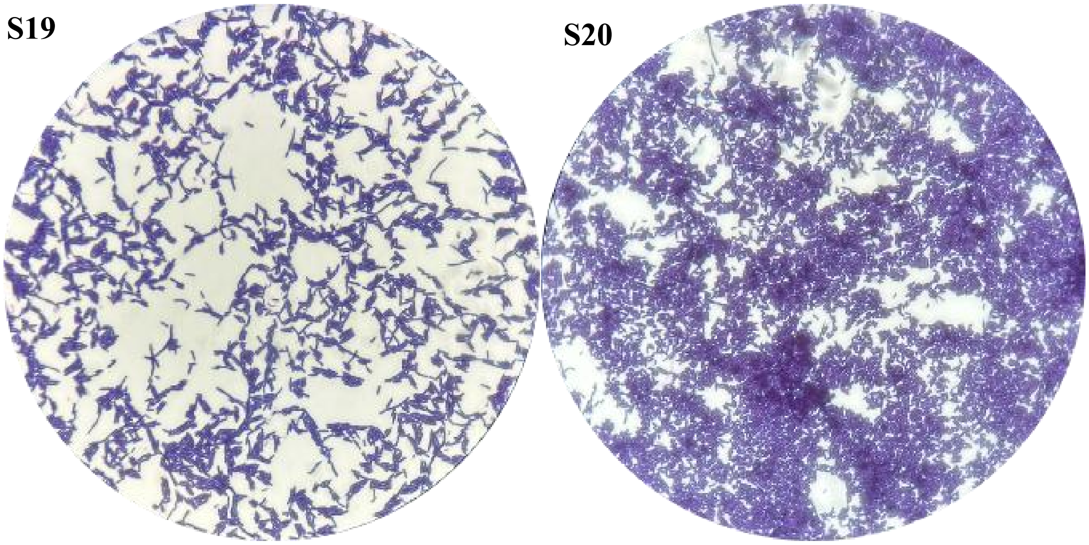

Supplementary Figure 2. The morphology of 20 isolated strains.

S5

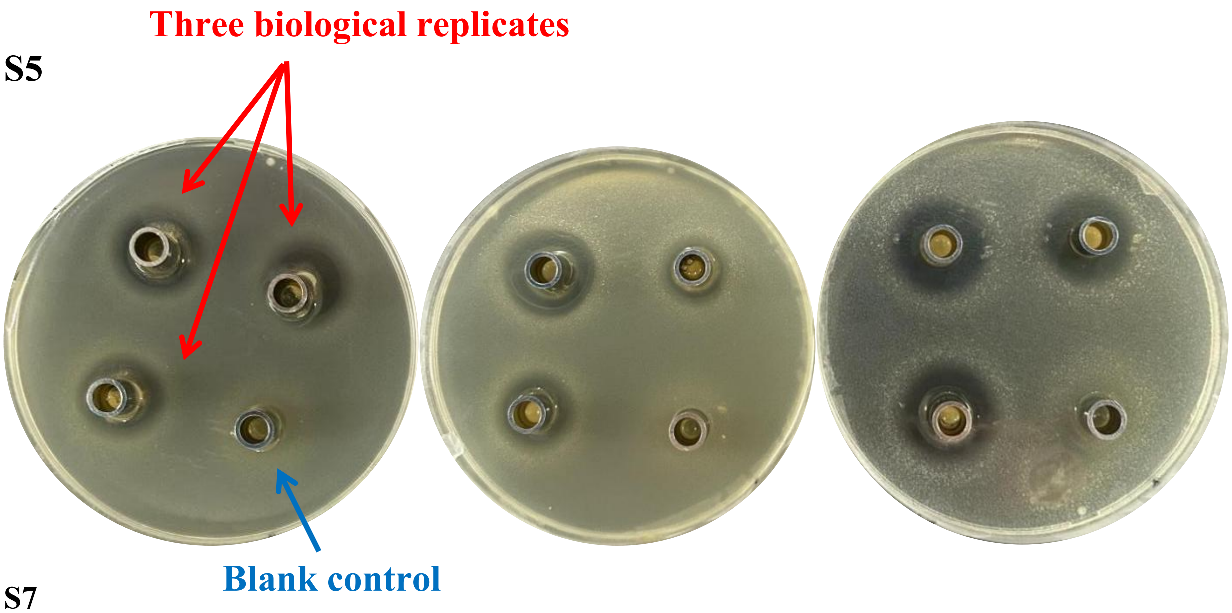

S7

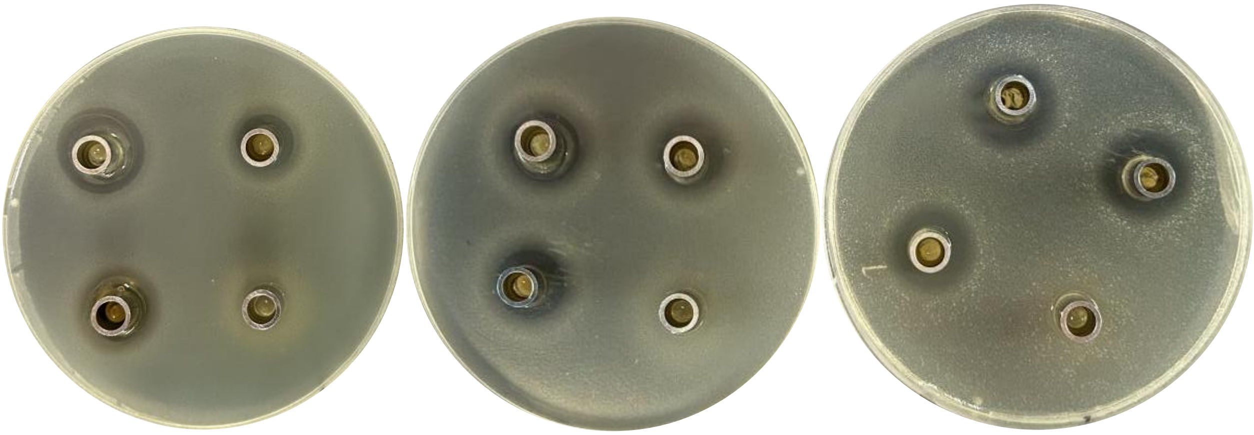

S10

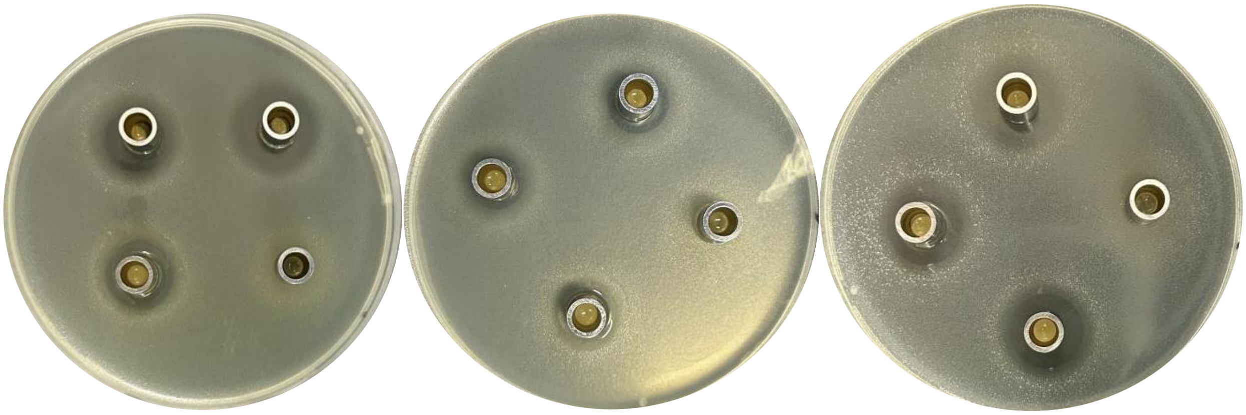

S11

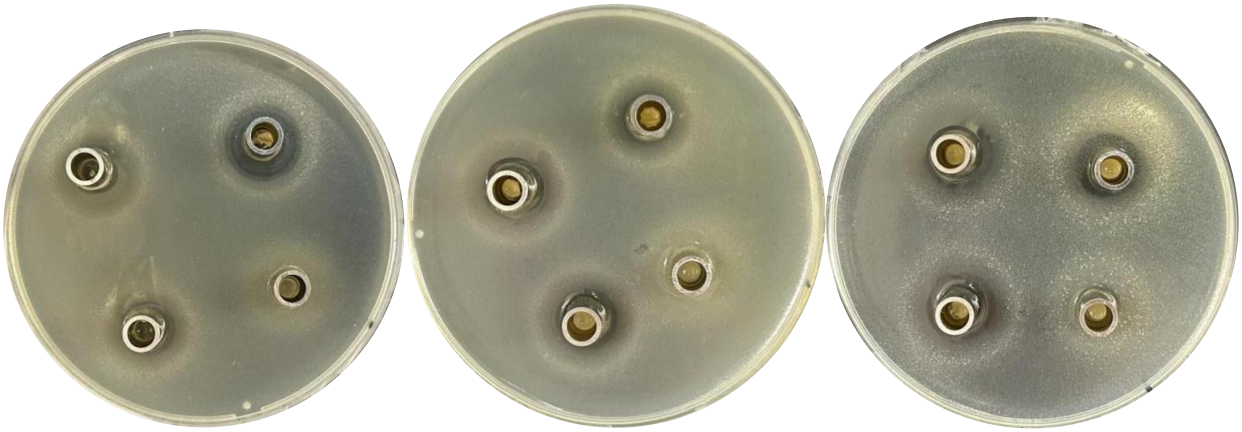

S19

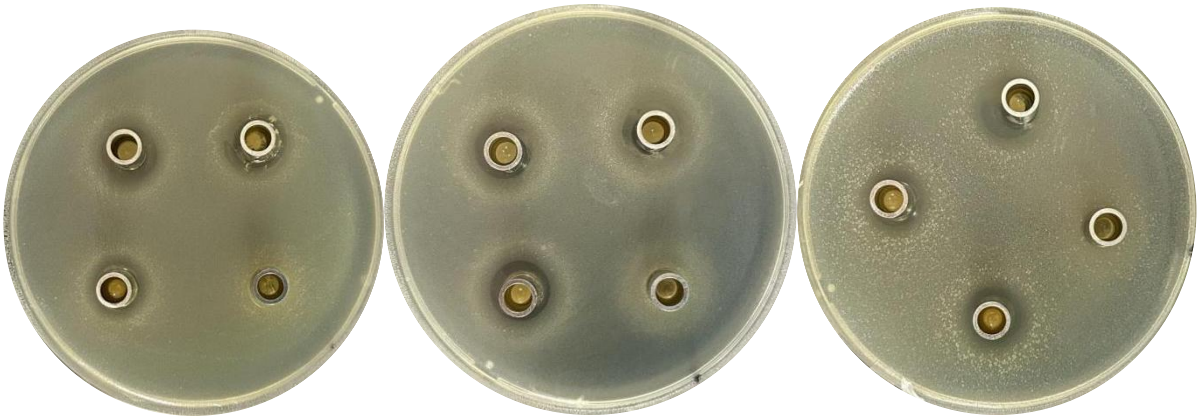

Supplementary Figure 3. The antibacterial activity of 5 isolated strains against different pathogenic bacteria (In each line, from left to right are *E. coli*, *Salmonella* and *S. aureus*).
